# Digital Education for Health Professionals: An Evidence Map, Conceptual Framework, and Research Agenda

**DOI:** 10.2196/31977

**Published:** 2022-03-17

**Authors:** Lorainne Tudor Car, Selina Poon, Bhone Myint Kyaw, David A Cook, Victoria Ward, Rifat Atun, Azeem Majeed, Jamie Johnston, Rianne M J J van der Kleij, Mariam Molokhia, Florian V Wangenheim, Martin Lupton, Niels Chavannes, Onyema Ajuebor, Charles G Prober, Josip Car

**Affiliations:** 1 Lee Kong Chian School of Medicine Nanyang Technological University Singapore Singapore; 2 Department of Primary Care and Public Health School of Public Health Imperial College London London United Kingdom; 3 Centre for Population Health Sciences Lee Kong Chian School of Medicine Nanyang Technological University Singapore Singapore; 4 Office of Applied Scholarship and Education Science, School of Continuous Professional Development Mayo Clinic College of Medicine and Science Division of General Internal Medicine, Mayo Clinic Rochester, MN United States; 5 Department of Pediatrics Lucile Packard Children’s Hospital Stanford, CA United States; 6 Department of Global Health and Population Harvard T.H. Chan School of Public Health Harvard Boston, MA United States; 7 Stanford Center for Health Education’s Digital MedIC Initiative Stanford School of Medicine Stanford University Stanford, CA United States; 8 Department of Public Health and Primary Care Leiden University Medical Centre Leiden Netherlands; 9 School of Population Health and Environmental Sciences Faculty of Life Sciences and Medicine King’s College London London United Kingdom; 10 Department of Management, Technology, and Economics ETH Zurich Zurich Switzerland; 11 Faculty of Medicine Imperial College London London United Kingdom; 12 The Chelsea and Westminster Hospital Chelsea London United Kingdom; 13 Health Workforce Department World Health Organization Geneva Switzerland

**Keywords:** digital education, health professions education, evidence map, systematic review, research questions, conceptual framework, mobile phone

## Abstract

**Background:**

Health professions education has undergone major changes with the advent and adoption of digital technologies worldwide.

**Objective:**

This study aims to map the existing evidence and identify gaps and research priorities to enable robust and relevant research in digital health professions education.

**Methods:**

We searched for systematic reviews on the digital education of practicing and student health care professionals. We searched MEDLINE, Embase, Cochrane Library, Educational Research Information Center, CINAHL, and gray literature sources from January 2014 to July 2020. A total of 2 authors independently screened the studies, extracted the data, and synthesized the findings. We outlined the key characteristics of the included reviews, the quality of the evidence they synthesized, and recommendations for future research. We mapped the empirical findings and research recommendations against the newly developed conceptual framework.

**Results:**

We identified 77 eligible systematic reviews. All of them included experimental studies and evaluated the effectiveness of digital education interventions in different health care disciplines or different digital education modalities. Most reviews included studies on various digital education modalities (22/77, 29%), virtual reality (19/77, 25%), and online education (10/77, 13%). Most reviews focused on health professions education in general (36/77, 47%), surgery (13/77, 17%), and nursing (11/77, 14%). The reviews mainly assessed participants’ skills (51/77, 66%) and knowledge (49/77, 64%) and included data from high-income countries (53/77, 69%). Our novel conceptual framework of digital health professions education comprises 6 key domains (context, infrastructure, education, learners, research, and quality improvement) and 16 subdomains. Finally, we identified 61 unique questions for future research in these reviews; these mapped to framework domains of education (29/61, 47% recommendations), context (17/61, 28% recommendations), infrastructure (9/61, 15% recommendations), learners (3/61, 5% recommendations), and research (3/61, 5% recommendations).

**Conclusions:**

We identified a large number of research questions regarding digital education, which collectively reflect a diverse and comprehensive research agenda. Our conceptual framework will help educators and researchers plan, develop, and study digital education. More evidence from low- and middle-income countries is needed.

## Introduction

The world is faced with a shortage and an unequal distribution of health workforces across low-income, middle-income, and high-income countries [1]. The shortfalls and inequitable distributions affect the likelihood of reaching the United Nations’ third Sustainable Development Goal—health and well-being for all by 2030 [1-5]. To enable an increase in and a more equitable distribution of competent health workforce, there is a need for more effective and accessible health professions education.

The use of digital technology in health professions education can help in overcoming some of the health workforce–related challenges by providing more accessible, standardized, relevant, timely, and affordable medical education and training [6,7]. Until recently, digital education was perceived as primarily supporting in-person health professions education [8]. The social distancing measures introduced to control the COVID-19 pandemic have dramatically changed the delivery of health professions education worldwide. Many medical schools and health professions education institutions had to pivot to digital education [9,10]. With this sudden shift, research and evidence in digital health professions education have become even more important.

The evidence on digital education has grown substantially in recent years and has been the subject of many systematic reviews. Existing reviews seem to mostly focus on the effectiveness of different digital education modalities [11-16]. However, the adoption of digital education is complex and includes other research questions, in addition to its effectiveness. It is important to identify evidence that already exists and evidence gaps across the full scope of relevant questions to inform and guide future research and reduce research waste. To address this need, we seek to (1) create a map of existing research, (2) develop a conceptual framework outlining key components of digital education, and (3) highlight specific research questions across a comprehensive research framework. We do this by systematically identifying and analyzing previous systematic reviews on digital health education.

## Methods

We used an evidence map methodology to identify and summarize systematic reviews on digital health professions education [17]. We also developed a novel conceptual framework using an established methodological approach [18] and identified specific research questions in alignment with this conceptual framework.

### Study Identification

To identify relevant systematic reviews on different types of digital health education for health professionals, we used a comprehensive search strategy mentioned in Multimedia Appendix 1, including key terms for participants (eg, *health professionals*, *health personnel*, and *students*), intervention (eg, *e-learning*, *patient simulation*, and *serious games*), and article type (eg, *systematic reviews* and *evidence synthesis*). We searched the following major bibliographic databases for studies published between January 1, 2014, and July 21, 2020, without any restrictions on language or study design: MEDLINE (Ovid), Embase (Ovid), Cochrane Library, Educational Research Information Center (EBSCO), and CINAHL (EBSCO). We also manually checked the reference lists of the included systematic reviews for other potentially relevant systematic reviews on digital education for health professionals. In addition, we searched Google, Google Scholar, ResearchGate, and OpenGrey for any other studies that might be relevant for our topic, using keywords *systematic review*, *digital education*, *health professionals*, *health professions education*, *eLearning*, and *e-learning*, and reviewing either the first 10 pages or 500 results.

### Eligibility Criteria

We included systematic reviews focusing on digital education for health care professionals in preservice (ie, student) and in-service (ie, postdegree, including postgraduate trainees and those in independent practice) positions [19]. This includes disciplines such as medicine, dentistry, nursing and midwifery, medical diagnostic and treatment technology, physiotherapy and rehabilitation, and pharmacy. Practitioners of traditional, alternative, and complementary medicine were excluded. Digital health professions education refers to health professions education that is conducted using digital technology [20] and includes modalities ranging from the basic conversion of content into a digital format (eg, a book converted into a PDF or HTML format) to more complex applications such as mobile education, digital games, virtual patients, and virtual reality (VR). Systematic reviews were included if they focused on ≥1 modality of digital education (as defined in Textbox 1) delivered in a stand-alone or blended format [20]. We defined blended education as education that incorporates aspects of traditional and digital education. Traditional education was defined as education that encompasses the use of nondigital educational materials (eg, textbooks or models) or in-person human interactions. We included systematic reviews of all the types of studies. We excluded older reviews because of the rapid evolution of the field, with the assumption that most of the active research questions from the reviews published >5 years ago would be collated in more recent reviews.

**Digital education technologies and modalities and working definitions and descriptions.**

**Offline digital education**

- Education delivery requires no internet or local area network connection and can be delivered through external media, including CD-ROM, external hard disk, and USB stick [21].

**Online digital education**

- Computer-assisted instruction using the internet or a local intranet as the means of delivery, also referred to as online, internet-based, or networked [22,23], includes multiple media formats (eg, text, videos, and images and online discussion (eg, via email, chat, or videoconferencing) and is designed to be primarily delivered on PCs.

**Massive open online course**

- A (free) online course available over the internet to a large number of geographically dispersed participants [24]

**Mobile education (m-Learning)**

- Flexible and accessible learning delivered via personal mobile devices, such as smartphones and tablets [25]

**Serious gaming and gamification**

- Knowledge and training activities are set within a competitive activity. Games are intended to promote the development of knowledge, cognitive skills, or psychomotor skills in a virtual environment [26].

**Virtual reality**

- Interactive exploration of a digital (3D) multimedia environment can reflect a real-world environment (eg, clinic) or an artificial or surreal context (eg, positioning the learner within the human body) [16,27].

**Virtual patient**

- A computer program that simulates real-life clinical scenarios where students take on the role of a health professional and obtain a patient’s history, conduct a physical examination, and make diagnostic and therapeutic decisions [28]

**High-fidelity manikins**

- Realistic, computerized mannequins that mimic elements of human physiology (eg, breathing and heart rhythm) and are used to simulate a real-life clinical scenario [29].

**Blended education**

- The use of digital education modalities in combination with traditional education methods

**Traditional education**

- Education that uses nondigital educational material (eg, textbook or model) or in-person human interaction

### Study Selection

The search results from different databases were combined in a single EndNote library, and duplicate citations were removed. A total of 2 review authors (SP and BMK) screened all titles and abstracts for inclusion independently and in duplicate. Disagreements during the title and abstract screening were resolved by consensus. Full texts of articles considered eligible or uncertain based on the title and abstract screening were retrieved and screened independently and in duplicate by the same 2 authors.

### Data Extraction

From the included systematic reviews, 2 authors (SP and BMK) used a standardized form to independently extract information on the review aim; the study design, participants, interventions, comparators, and outcomes of the original research studies included; the method used to appraise the quality of the included studies (eg, risk of bias) or overall evidence (eg, the Grading of Recommendations Assessment, Development and Evaluation assessment); and recommendations for future research. We classified the outcomes according to the definitions presented in Multimedia Appendix 2. We also extracted information on all additional outcomes reported in the included reviews that did not correspond to our predefined outcome-related definitions. We classified the systematic review in terms of the single digital modality they most focused on, according to our framework (Textbox 1). In most instances, it was clear that a given review focused predominantly on 1 modality. Less often, a review encompassed multiple modalities equally, in which case we classified it as digital education; that is, the use of digital technology in health professions education in general. Finally, we identified recommendations for future research by extracting exact quotes from each review that articulated such recommendations. At every stage, disagreements between the review authors were resolved through discussion and input from the third author (LTC).

### Analysis

Authors SP and BMK rephrased the quoted research recommendations into research questions and then refined these by applying consistent terminology, removing duplicates, and merging or subdividing themes. Questions that focused on specific digital modalities (eg, online modules) were rewritten to make them relevant to the broader research agenda for digital health education. The final list of research questions was refined by LTC.

We developed a conceptual framework outlining various digital health professions’ education components according to the methodology described by Jabareen [18]. We consulted and built on our previous conceptual work in this area and existing frameworks for the implementation or adoption of digital education generally [30-39]. We identified these frameworks through a focused literature search on PubMed, Google, and Google Scholar. On the basis of the discussions and consensus among the review authors, key domains and subdomains were finalized. The framework and its components were represented diagrammatically. Recommendations for future research were classified according to the proposed framework in parallel by 2 authors (SP and BMK). Discrepancies were resolved through consensus and with the guidance of the third author (LTC). On the basis of the analysis of the included reviews and the observed gaps in the literature, we outline a research agenda for digital health professions education and present it in the *Results* section.

## Results

### Search Results

**Figure 1:**
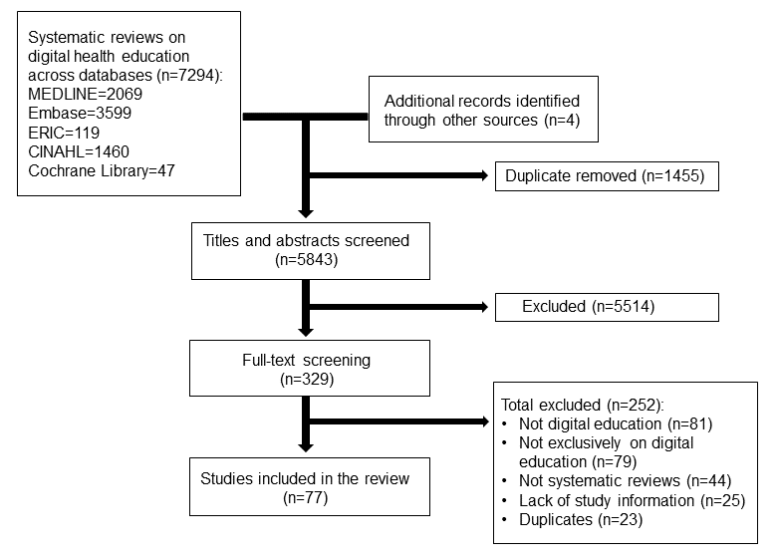
Study flow diagram. ERIC: Educational Research Information Center.

Of 7294 systematic reviews from our initial search, we identified 73 (1%) eligible systematic reviews (Figure 1). Another 4 systematic reviews were identified through Google, Google Scholar, ResearchGate, and OpenGrey. In total, 77 systematic reviews were included for data extraction.

The number of published systematic reviews increased over time, from 6% (5/77) published in 2014 to 56% (43/77) published in 2019. The systematic reviews focused on digital education for health professions students (17/77, 22% studies), postgraduate or independently practicing (18/77, 23% studies) health professionals, or both (42/77, 55% studies). Most of the systematic reviews were on digital education in surgery (13/77, 17% studies) or health professions education in general (ie, those not focusing on a particular type of practitioner, health care area, or topic; 36/77, 47% studies), followed by nursing (11/77, 14% studies; Table 1; Multimedia Appendix 3 [11-16,32,34,40-109]).

**Figure 2:**
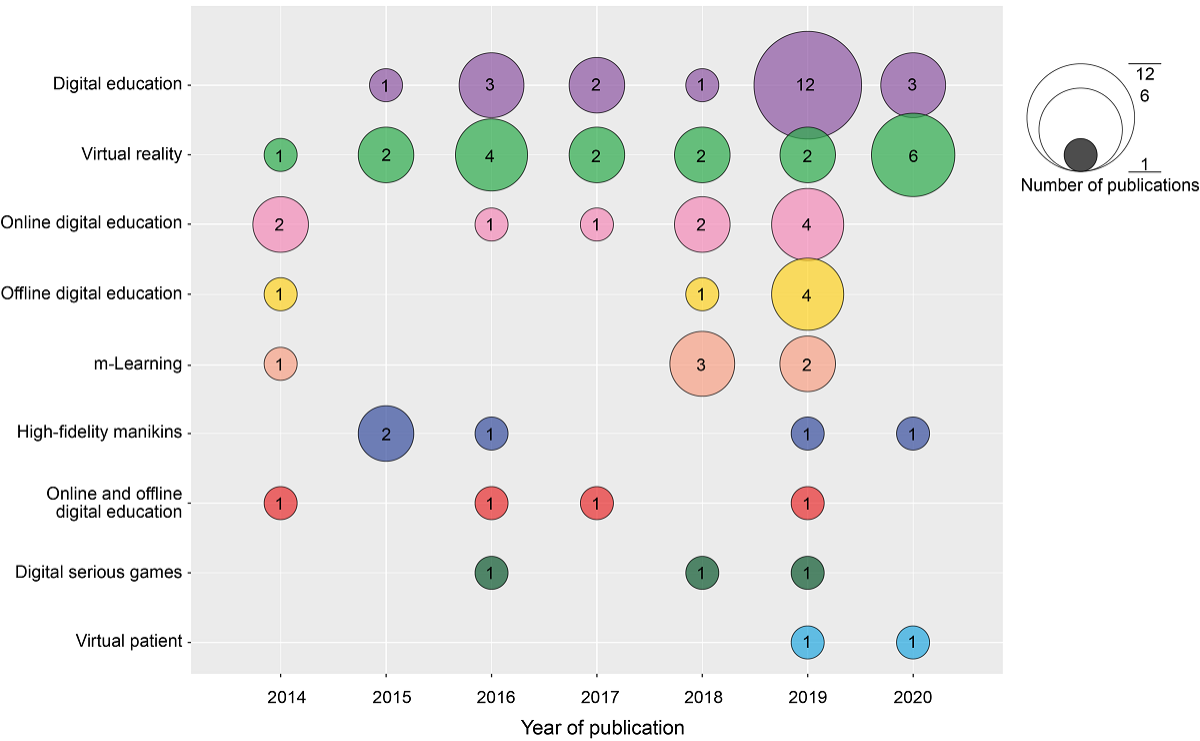
The number of systematic reviews on different digital modalities according to the year of publication. m-Learning: mobile learning.

A breakdown of the digital modalities being investigated in the included systematic reviews is shown in Figure 2. Most systematic reviews focused on digital education in general (22/77, 29%), VR (19/77, 15%), and online education (10/77, 13%). Of the 19 reviews on VR, 17 (89%) were on VR complemented with physical objects or devices such as probes or handles and focused on psychomotor, procedural, or technical skill development. There were fewer reviews published on m-Learning (6/77, 8%), digital game-based learning (3/77, 4%), and virtual patients (2/77, 3%).

The most common outcomes in the included reviews were health professionals’ knowledge (49/77, 64%), skills (51/77, 66%), attitudes about the clinical topic (13/77, 17%), and satisfaction with digital education (18/77, 23%). Most systematic reviews compared the effectiveness of digital education to traditional education (ie, nondigital; 59/77, 77%) or other forms of digital education (35/77, 45%; Table 1). Most reviews reported only immediate, short-term outcomes; 22% (17/77) of reviews reported the impact of digital education on long-term delayed outcomes; that is, outcomes assessed with delay after the intervention [34,40-55]. Most reviews appraised methods using the Risk of Bias tool [110] only (24/77, 31%), followed by Grading of Recommendations, Assessment, Development, and Evaluation [111] (which also includes the risk of bias assessment; 22/77, 29%) and Medical Education Research Study Quality Instrument [112] (10/77, 13%). Of the 77 studies, 9 (12%) reviews did not report on the quality appraisal of the included evidence, whereas the remaining 14 (18%) reviews used different instruments to determine the evidence quality (Table 1; Multimedia Appendix 3 [11-16,32,34,40-107,109]). The included reviews mostly reported original research to be low or very low quality of evidence or reported unclear or high risk of bias in most studies (Multimedia Appendix 3 [11-16,32,34,40-107,109]). Most systematic reviews reported data from high-income countries (14/77, 18% systematic reviews) or both middle- and high-income countries (42/77, 55% systematic reviews). Only 4% (3/77) of the systematic reviews included studies from low-income countries [11,56,57]. Approximately 29% (22/77) of the included reviews did not report the setting of the included studies.

**Table 1:** Characteristics of the included systematic reviews (N=77).

Characteristics of the systematic reviews and the evidence they include		Studies, n (%)
**Type of participants**		
	Medical students	5 (6)
	Medical students and physicians	9 (12)
	Physicians	17 (22)
	Dentistry students	3 (4)
	Dentistry students and dentists	2 (3)
	Nursing students	8 (10)
	Nursing students and nurses	3 (4)
	Mixed students	2 (3)
	Mixed students and HCPs^a^	19 (25)
	Mixed HCPs	9 (12)
**Level of education**		
	Postdegree: practicing HCPs	10 (13)
	Postdegree: trainees^b^	5 (6)
	Postdegree: mix of trainees^b^ and practicing HCPs	3 (4)
	Student	17 (22)
	Mixed student and postdegree	42 (55)
**Clinical topics**		
	General health professions education	23 (30)
	Surgery	14 (18)
	Nursing	8 (10)
	Life support or trauma management (resuscitation skills)	3 (4)
	Radiology	7 (9)
	Endoscopy	3 (4)
	Other	19 (25)
**Setting**		
	High-income countries only	26 (34)
	High-income and middle-income countries	45 (58)
	High-, middle-, and low-income countries	4 (5)
	Middle- and low-income countries	1 (1)
	Information not available	1 (1)
**Modality**		
	Digital education	22 (29)
	Virtual reality	19 (25)
	Online	10 (13)
	Offline	6 (8)
	Mobile learning	6 (8)
	High-fidelity manikins	5 (6)
	Online and offline	4 (5)
	Digital serious games	3 (4)
	Virtual patient	2 (3)
**Comparison**		
	No intervention	25 (32)
	Traditional education	56 (73)
	Digital intervention	35 (45)
	Other	6 (8)
**Quality appraisal**		
	Risk of bias	24 (31)
	Grading of Recommendations, Assessment, Development, and Evaluations	22 (29)
	Medical Education Research Study Quality Instrument	10 (13)
	Best Evidence in Medical Education reviews	2 (3)
	The Jadad scale	2 (3)
	Joanna Briggs Institute Meta-Analysis of Statistics Assessment and Review Instrument	2 (3)
	Methodological index for non-randomized studies	1 (1)
	Newcastle–Ottawa Scale	1 (1)
	Other	5 (6)
	Not reported	8 (10)
**Outcomes**		
	Knowledge	49 (64)
	Skills	51 (66)
	Satisfaction	18 (23)
	Patient outcomes	20 (26)
	Performance^c^	19 (25)
	Attitude	13 (17)
	Behavioral	8 (10)
**Number of included studies in the review**		
	<10	24 (31)
	10-19	27 (35)
	20-29	10 (13)
	30-39	8 (10)
	≥40	8 (10)
**Study designs included in the review**		
	Randomized controlled trials	68 (88)
	Other experimental studies^d^	8 (10)
	Cross-sectional studies	5 (6)
	Qualitative studies	3 (4)
	Pre-post studies	12 (16)
	Cohort studies	8 (10)
	Other or mixed^e^	34 (44)
**The conceptual framework domain or subdomain the reviews focus on**		
	Education—design	77 (100)
	Education—content	77 (100)
	Education—evaluation	9 (12)
	Education—pedagogy	5 (6)
	Education—engagement	3 (4)
	Context—settings	1 (1)

^a^HCP: health care professional.

^b^Includes residents, novices, trainees, and fellows.

^c^Defined in the included systematic reviews as a combination of skills and behavioral changes as a result of the intervention.

^d^Includes quasi-randomized controlled trials, nonrandomized controlled trials, before-and-after studies, and interrupted time series designs.

^e^Includes study designs not described above or a combination of different study designs.

### Conceptual Framework of Digital Health Professions Education

**Figure 3:**
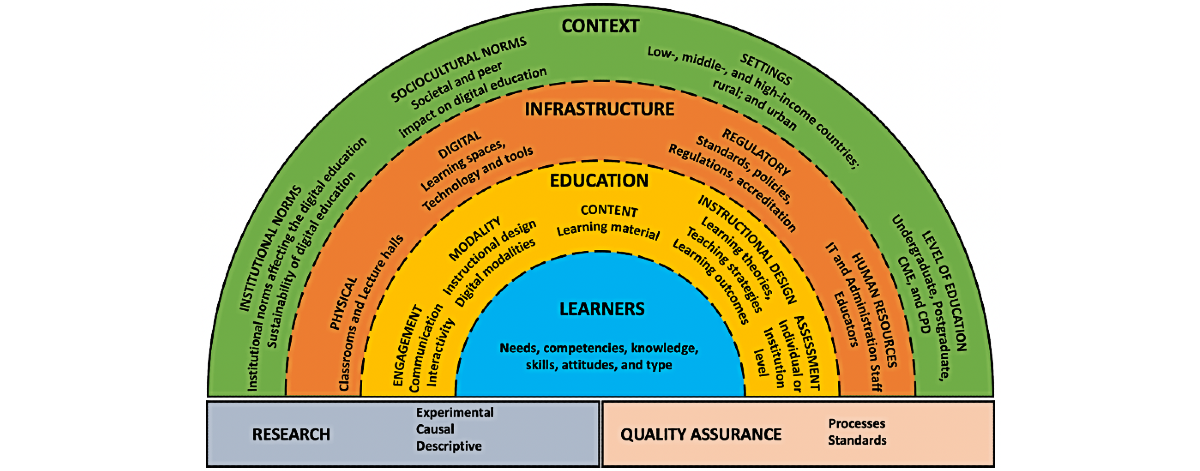
Conceptual framework of digital health education for healthcare professionals. CME: continuing medical education; CPD: continuing professional development; IT: information technology.

To outline different aspects of digital health professions education and identify gaps in the literature, we developed a novel conceptual framework (Figure 3) grounded in key findings of these systematic reviews together with 7 existing frameworks for digital education general [35-39,113-117] and a framework we developed previously for health professions education [33].

Broadly, the fundamental domains include an enabling and supportive *context*, sound *infrastructure,* and the optimal use of *education* tools and processes. The context is a combination of institutional norms, sociocultural norms, and settings in which the learner resides, as well as the level of education the learner is at. Subdomains of the context have a direct impact on the infrastructure components required and available for the delivery of digital education—digital and physical spaces, policies and regulatory standards, and human resources. Both context and infrastructure components are important in consideration of health professions education. *Learners*, individually and as part of a larger group, are at the core of digital health education, and their needs, preferences, prior expertise, and competencies should shape how education is delivered. The interaction among components within and across each layer is dynamic, with different parts being interconnected, as reflected using dotted lines to separate context, infrastructure, education, and learners. Studying and identifying optimal relationships between the components are handled by the *research* and *quality assurance* blocks, which are connected to the rest of the framework. Table 2 provides the detailed operational definitions for each domain of the framework.

**Table 2:** Definitions of digital health professions education conceptual framework components.

Domain and subdomain		Definition
**Context**		
	Sociocultural norms	The acceptability and adoption of digital education as a form and norm of education within the society
	Institutional norms	The acceptability, impact, considerations, and processes concerning the adoption of digital education at the institutional level
	Settings	The setting in which digital health education is conducted or implemented, including clinical or classroom environments; low-, middle-, and high-income countries; and rural or urban environments
	Level of education	The impact and integration of digital education with other forms of education (eg, inter- and intraprofessional training opportunities) and clinical work in which participants are engaged
**Infrastructure**		
	Physical	The physical learning space within which the in-person component of blended digital health education is taking place
	Digital	The information and communication technology devices (both hardware and software) to support and create learning environments (virtual environments, digital networks, technological modifications) or media for digital health education, as well as the speed and capacity of internet access
	Regulatory	Policies and regulatory standards for health professionals’ licensing and accreditation, as well as those relating to the design and delivery of digital health professions education
	Human resources	The human resources required for digital health education to be maintained and sustained, including educators, administrators, and information technology staff
**Education**		
	Modality	The choice and configuration of digital education modality (eg, online learning and m-Learning) and its potential blending with in-person education
	Instructional design	The method and practice of digital health professions education encompassing teaching strategies, learning principles, learning outcomes, and the assessment approach
	Content	Health professions education area, discipline, theme, or topic delivered via digital education
	Engagement	The level of communication, interactivity, or immersion of participants taking part in digital health professions education
	Assessment	Measurement of digital health professions education conducted at the individual and institutional level to determine its impact on educational and clinical outcomes
Learners		Health professionals with distinctive needs, competencies, digital literacy, knowledge, skills, and attitudes toward working and learning, both individually or as a group
Research		Systematic study of digital health professions education to create and disseminate new knowledge and allow for more effective and efficient adoption, implementation, and transfer of interventions to various contexts—this encompasses experimental, observational, descriptive, and qualitative research
Quality assurance		A context-specific and systematic evaluation of practices and procedures to understand the current state and improve the performance of digital health education in a particular setting

### Research Questions From the Included Systematic Reviews

We identified 318 discrete research questions posed in these 77 articles, from which we distilled a final list of 61 (19.2%) distinct questions covering 14 of the 16 subdomains of the above framework (Table 2; Multimedia Appendix 4 [11-16,32,34,40-95,98-107]). Research questions that spanned multiple subdomains were assigned by investigator consensus to the most relevant single subdomain. None of the included systematic reviews posed questions primarily directed at the *physical* infrastructure or *quality assurance* subdomains. We identified 26% (16/61) of questions relating to context, classified into four subdomains: *sociocultural norms*, *institutional norms*, *settings,* and *level of education*. Approximately 15% (9/61) of research questions (3 per subdomain) were identified for *the digital*, *regulatory,* and *human resources* subdomains within the infrastructure domain. Most of the research questions, 48% (29/61) approximately, were categorized in the education domain, which encompasses *modality, instructional design*, *content*, *engagement,* and *evaluation* subdomains. Approximately 5% (3/61) of *research* questions each were categorized in the *learners* and *research* domains.

### Classifying Research Questions Addressed by Existing Systematic Reviews

We also classified the included systematic reviews based on their research questions and using our conceptual framework (Table 3; Multimedia Appendix 3 [11-16,32,34,40-107,109]). The research questions addressed by existing systematic reviews mostly revolved around digital education *modality* (ie, the effectiveness of various digital education modalities delivered as stand-alone or blended interventions) and *content* (ie, the effectiveness of digital education within a particular health care area or discipline). Some reviews assessed interactivity (*engagement*), various aspects of *instructional design* in digital education, the impact of digital education on institutional outcomes (*context—institutional norms*), and the impact of *setting* (eg, low-income and middle-income countries) on learning outcomes.

**Table 3:** Research questions identified from the included systematic reviews on health professions digital education.

Research questions identified from included systematic reviews	Conceptual framework domain (subdomain)	Systematic reviews’ references
How do cost and cost-related outcomes influence the adoption of digital technology in health professions education?	Context (sociocultural norms)	[41,56,58-65]
How can policy makers be organized to adopt digital education as part of health professions education?	Context (sociocultural norms)	[56,66]
How do cultural factors within different countries determine the use of digital education for health professions training?	Context (sociocultural norms)	[66]
How does providing access to digital education improve the learning outcomes of health professionals?	Context (sociocultural norms)	[14,40,41,43,46,53,56,57,66-77]
What is the long-term cost-effectiveness of digital education compared with traditional education for health professionals?	Context (institutional norms)	[12-14,16,47,61,78]
How does health professions’ digital education affect individual and health services outcomes and organizational practice?	Context (institutional norms)	[11-14, 16, 32, 34, 40, 42-45, 48, 49, 51-53, 56, 60, 62, 64, 67-70, 75, 79-88]
Is health professions’ digital education more time efficient than traditional education?	Context (institutional norms)	[46]
What is the feasibility of implementing digital technology for health professions education in different socioeconomic settings?	Context (setting)	[13,14,16,43,56,57,78,89,90]
What are the short- and long-term effects of using digital technology for health professions education in different socioeconomic settings?	Context (setting)	[32,43,47,50,54,60,78,82,89-91]
Is digital education for health professionals effective in different socioeconomic settings?	Context (setting)	[13,40,91,92]
What are the resource requirements to implement digital education in different socioeconomic settings?	Context (setting)	[85,93]
What are the challenges of setting up digital education for health professionals training in different socioeconomic settings?	Context (setting)	[85]
What is the differential impact of digital education on the clinical performance of trainee or expert surgeons?	Context (level)	[94]
How can digital education for health professionals be integrated into normal work practices?	Context (level)	[68]
How can digital technology be incorporated into current health professions’ education and training curriculum to improve learning outcomes?	Context (level)	[42,46,47,54,61,62,78,90]
Is digital education effective in improving health professionals’ knowledge and skills performance in the clinical setting?	Context (level)	[11-14, 16, 32, 34, 40, 42-45, 48, 49, 51-53, 56, 60, 62, 64, 67-70, 75, 79-88]
Which features of digital education (eg, technical features, fidelity, safety, and adaptability) affect the learning outcomes of health professions education?	Infrastructure (digital)	[13,95]
What are the minimum requirements for the digital technology used to achieve the effectiveness of digital health professions education?	Infrastructure (digital)	[85]
What are the technical resources needed to deliver digital education to health care professionals?	Infrastructure (digital)	[61]
How should educators delivering digital health education be assessed and accredited?	Infrastructure (regulatory)	[47]
What are the best practices for the development, evaluation, and use of digital health education in health professions education?	Infrastructure (regulatory)	[14]
Is the use of accreditation-related milestones in digital health education effective?	Infrastructure (regulatory)	[78]
What digital skills should instructors facilitating digital health education be competent in?	Infrastructure (human resources)	[47]
How does the digital competence of teachers affect health professions learning outcomes from digital health education?	Infrastructure (human resources)	[96]
What are the workforce resources needed for health professions’ digital education?	Infrastructure (human resources)	[61]
What type of instructional design is used in the effective digital education of health professions education?	Education (modality)	[47,57,83,87,94]
Which components of digital health education (eg, interactivity and feedback) contribute to enhanced learning outcomes?	Education (modality)	[45,52,58,67,97]
What is the optimal use of video-assisted debriefing for health professionals’ simulation-based training?	Education (modality)	[98]
How does the design of digital education interventions (eg, format and modality used) in health professions education and training curriculum affect learning outcomes?	Education (modality)	[34,42,53,64,74,78,93]
Can digital simulation-based training be used to train nontechnical skills in health professionals?	Education (modality)	[44,69]
What is the effectiveness of digital education (mixed or single modality) compared with nondigital education to deliver health professions education?	Education (modality)	[42,71,98]
Can digital education complement (ie, blended) or substitute traditional education for health professionals?	Education (modality)	[54,69,99,100]
Does digital simulation-based psychomotor skills training provide any benefit to the medical trainee?	Education (modality)	[46]
What are the barriers to obtaining digital education materials for health professions education training, and how can they be overcome?	Education (content)	[66]
What content should be included in debriefing (eg, digital data) following simulation-based education to achieve improved clinical outcomes?	Education (content)	[47]
Can digital education be used to overcome challenges in delivering content-specific topics for health professions education (eg, surgical training in rare pathologic states)?	Education (content)	[84,89]
Can digital education be designed to achieve learning outcomes denoted in the Kirkpatrick model?	Education (instructional design)	[101]
What learning theories can be used to inform the development of effective digital health professions education?	Education (instructional design)	[13,14,55,59,63,82,93]
Is mastery learning via digital education more or as effective as traditional education in terms of clinical psychomotor skills improvement?	Education (instructional design)	[41,47,48,53,58-60,78,102]
Is spacing digital simulation–based training more or as effective as traditional education in clinical psychomotor skills development?	Education (instructional design)	[41,47,48,53,58-60,78,102]
How does the frequency and duration of digital simulation–based psychomotor skills training affect health professionals’ skills transfer to the clinical setting?	Education (instructional design)	[41,47,48,53,58-60,78,102]
What are the optimal duration, frequency, and intensity of digital health professions education programs to affect the learning and clinical outcomes of health professionals?	Education (instructional design)	[43,54,62,72,83,94,103]
What pedagogy should be used in the digital education of health professionals to improve their knowledge and skills?	Education (instructional design)	[11,14,42,95,104]
What is the effectiveness of using digital education to train and assess nontechnical skills in health care professionals?	Education (instructional design)	[71,87]
What is the effectiveness of digital problem–based learning in health professions education?	Education (instructional design)	[34]
How does the interactivity of digital education programs affect the learning and clinical outcomes of health professionals?	Education (engagement)	[53,62,80,91]
What is the minimal level of haptic feedback required in digital simulation-based training programs to improve health professionals’ psychomotor skills?	Education (engagement)	[64]
What are learners’ acceptability of digital education with different levels of interactivity?	Education (engagement)	[77]
Which performance metrics or measurement instrument should be used to assess health professionals’ knowledge, skills, attitudes, satisfaction, and clinical outcomes from digital technology–based training programs?	Education (assessment)	[12, 14, 44, 45, 51-53, 60, 62, 64, 67-71, 73-75, 77, 78, 83, 87, 90, 92, 93, 95, 102, 103, 105]
What is the ideal approach to assessing health professionals’ knowledge, skills, attitudes, satisfaction, and clinical outcomes from digital technology–based education and training programs?	Education (assessment)	[12, 14, 44, 45, 51-53, 60, 62, 64, 67-71, 73-75, 77, 78, 83, 87, 90, 92, 93, 95, 102, 103, 105]
Should the evaluation of digital health education include behavior and clinical outcomes?	Education (assessment)	[12, 14, 44, 45, 51-53, 60, 62, 64, 67-71, 73-75, 77, 78, 83, 87, 90, 92, 93, 95, 102, 103, 105]
What is the impact of digital simulation–based training on clinical outcomes in the short and long term?	Education (assessment)	[71,106]
How should learning outcomes in the field of digital health professions education be defined and standardized?	Education (assessment)	[92]
How does the use of digital education affect health professionals’ clinical decision-making at the point of care?	Education (assessment)	[68]
How do health professionals’ prior learning experiences influence the topics that will benefit from the use of digital education?	Learner	[107]
What are health professionals’ attitudes toward digital delivery of education and training programs?	Learner	[16,65,85,91]
What are health care professionals’ learning needs, and can they be met by the use of digital simulation training?	Learner	[44]
What are the methodological requirements for studies assessing digital health education?	Research	[12, 16, 48, 53, 58, 59, 63, 65, 66, 70, 71, 74, 82, 83, 92, 95, 97]
How should studies on digital health professions education be reported?	Research	[12, 16, 48, 53, 58, 59, 63, 65, 66, 70, 71, 74, 82, 83, 92, 95, 97]
How should studies of digital health professions education be designed to ensure the generalizability of their findings across different settings?	Research	[12, 16, 48, 53, 58, 59, 63, 65, 66, 70, 71, 74, 82, 83, 92, 95, 97]
What are the barriers and facilitators that affect the continued adoption of digital tools in health professions education?	Context, education, infrastructure, and learner	[68]

## Discussion

### Principal Findings

We present an evidence map of 77 systematic reviews on digital education for health professionals published between 2014 and July 2020. The reviews mostly focused on the effectiveness of various digital education modalities in surgery, health professions education in general, and nursing. Most reviews have focused on online and offline learning. Only a few reviews focused on other digital education modalities such as m-Learning, VR, digital game–based learning, and virtual patients. We developed a novel conceptual framework outlining key components of digital health professions education, namely context, infrastructure, education, learner, research, and quality assurance. Within these reviews, we identified 61 unique recommendations (questions) for future research, focusing primarily on digital education modality, instructional design, and assessment.

### Limitations and Strengths

Our study has some limitations. First, to cover the most recent evidence in the field of digital education for health care professionals, we excluded studies before 2014; earlier reviews might have identified important research questions that remain unanswered. Our focus on systematic reviews also excluded other article types, such as editorials or viewpoints, which might have identified additional research questions. Second, although extraction and classification of research questions were done in duplicate and using a standardized approach, other classifications could be justified in some instances, which implies a degree of imprecision in the reported frequencies of specific questions. Moreover, our method did not allow us to prioritize the numerous research questions; such prioritization would require input from a representative group of experts and could be the focus of a future study. Third, there are overlaps among different concepts specified within this conceptual framework, which could be delineated and presented differently depending on potential chosen emphasis or entry points. Fourth, reviews classified as *online education* varied substantially in their inclusion of other modalities (eg, some expressly excluded modalities such as virtual patients, digital games, or massive open online courses, whereas others included these and other modalities). Finally, our novel conceptual framework may require revision as our understanding of this field matures and evolves, additional evidence accumulates, and new technologies emerge.

Our study also has several strengths, such as a thorough literature search for relevant studies, encompassing several indexed and gray literature databases without restrictions. We followed an established evidence map methodology and performed the steps in duplicate and independently [17]. In the development of our conceptual framework, we drew from the existing frameworks, our previous work, and discussions with experts.

### Integration With Prior Work

We did not find other frameworks presenting a high-level overview of the use and implementation of digital education for health professionals. Therefore, we drew from the general digital education literature and found several relevant frameworks.(Multimedia Appendix 5). The included frameworks, while providing an overview of digital education, often had additional objectives such as exploring the role of specific stakeholders (eg, the private sector or the ministries), identifying barriers to adoption, or analyzing a particular digital education aspect, setting, or configuration [33,35-37,113-117]. Our framework complements other frameworks by pulling together domains previously presented only in isolation and by adding novel subdomains such as the impact of training levels, the role of regulations and accreditations, and the importance of physical infrastructure (Multimedia Appendix 4 [11-16,32,34,40-95,98-107]).

Several viewpoint articles have offered research agendas for digital health professions education [28,118-120]. They focus primarily on the design of interventions and research studies in this field, which correspond to the domains of education (modality, instruction design, assessment, and engagement), research (quality of methods and reporting), and context (setting and level of education) in our framework. The agendas espoused in these viewpoints include questions that probe more narrowly and deeply on specific issues relevant to the design and focus of future studies (eg, the choice of comparison intervention and avoidance of confounding, integration of digital education across different institutions, and the need for interdisciplinary collaboration). Our framework was intentionally broad and comprehensive and enabled us to accommodate a variety of additional questions on previously neglected topics.

### Implications for Research and Practice

Most reviews in our evidence map focused on the effectiveness of digital education interventions and rarely addressed issues around their implementation and adoption. These reviews also mostly compared the effectiveness of digital interventions with that of nondigital education. Findings from studies comparing digital and nondigital education have limited generalizability as these studies cannot account for variance within and between these 2 educational formats [118]. Future research should compare different digital education modalities as such studies are more likely to generate meaningful, generalizable findings. It should also aim to explore potential challenges related to the implementation and adoption of digital education interventions in different settings.

There is also a need for more methodologically robust research and clearer terminology in this field. The quality of the evidence, as reported in the included reviews, was relatively low, with a limited number of studies measuring skills and knowledge retention. Furthermore, it was at times difficult to determine which modality (or modalities) the included reviews focused on because of poorly explained inclusion criteria. Such ambiguity was particularly common in reviews on e-learning and blended, online, and internet-based education.

We also express concerns about the paucity of studies from low- and middle-income countries. Such countries could greatly benefit from digital education, especially by using free or low-cost education (eg, massive open online courses). Although some research findings have a universal application (eg, fundamental principles of effective learning), others (such as implementation, infrastructure, and learners) are more context specific. Given the presence of unique needs of low- and middle-income countries (eg, distinct content priorities, learner demographics, and infrastructure), we urge more research in these contexts.

Our conceptual framework will benefit researchers, funding agencies, and educators, among others. The specific questions identified and classified according to this framework provide a map for future research and can help prioritize original research studies and guide the planning of new or updated systematic reviews. We encourage investigators to broadly consider the questions we identified in this evidence map, especially those specific to areas previously less studied, such as infrastructure, learners, or quality assurance in digital education. Our framework can also be used by funding agencies to better understand the limitations of the existing research and identify areas with limited evidence with the aim of informing their funding calls in this field. Finally, this framework can encourage those developing new courses to anticipate and plan for issues that are important but might be inadvertently overlooked, such as the digital education context, infrastructure, and learners.

## Appendix

Multimedia Appendix 1 MEDLINE (Ovid) search strategy.

Multimedia Appendix 2 Educational outcomes reported in the included systematic reviews and their definitions.

Multimedia Appendix 3 Characteristics of the included systematic reviews.

Multimedia Appendix 4 Research questions identified in the included systematic reviews.

Multimedia Appendix 5 Overview of conceptual frameworks on the implementation and adoption of digital education.

## Abbreviations

VR: virtual reality
