# Supplementary material for: Digital Education for Health Professionals: An Evidence Map, Conceptual Framework, and Research Agenda
Source: J Med Internet Res. 2022 Mar 17;24(3):e31977. doi: 10.2196/31977 (PMC8972116; doi:10.2196/31977)
Supplement: Multimedia Appendix 1 [file jmir_v24i3e31977_app1.docx]

##### Appendix 1. MEDLINE search strategy

Search strategy for Medline (Ovid), and adapted for Embase (Ovid), Cochrane Library, Educational Research Information Centre (Ebsco) and Cumulative Index to Nursing and Allied Health Literature (Ebsco).

1 exp education, professional/ not education, veterinary/

2 Education, Predental/

3 Education, Premedical/

4 exp Students, Health Occupations/

5 ((medic* or premedic* or dent* or laborator* or predent* or midwi?e* or nurs* or nutrition* or orthop* or podiat* or pharmac* or psycholog* or psychiatr* or health or healthcare or occupational therap* or physiotherap* or physical therap* or clinical or surg* or radiolog* or obstetric* or gyn?ecolog* or orthodont* or An?esthesi* or Dermatolog* or Oncolog* or Rheumatolog* or Neurolog* or Patholog* or P?ediatric* or Cardiolog* or Urolog*) adj3 (student* or graduate* or undergraduate* or staff or personnel or practitioner* or clerk* or fellow* or internship* or residen* or educat* or train* or novice* or tutor*)).tw,kf.

6 or/1-5

7 Computer-Assisted Instruction/

8 exp Internet/

9 Computer Simulation/

10 Patient Simulation/

11 software/

12 Mobile Applications/

13 User-Computer Interface/

14 Video Games/

15 Web Browser/

16 Education, Distance/

17 Computers/

18 exp Microcomputers/

19 exp Cell Phones/

20 Games, Experimental/

21 exp Models, Anatomic/

22 Audiovisual Aids/

23 Educational Technology/

24 Electronic Mail/

25 exp Telemedicine/

26 Telenursing/

27 Telecommunications/

28 Webcasts/

29 exp Videoconferencing/

30 ((computer* or digital* or hybrid or blended or mixed mode or distance or remote* or electronic or mobile or online* or interactiv* or multimedia or internet or web* or virtual* or game* or gaming or Videogame* or Videogaming) adj3 (classroom* or course* or educat* or instruct* or learn* or lecture* or simulat* or train* or teach* or tutor* or platform*)).tw,kf.

31 (Simulat* adj3 (course* or educat* or instruct* or learn* or train* or teach* or platform* or high-fidelity)).tw,kf.

32 e-learn*.tw,kf.

33 elearn*.tw,kf.

34 m-learn*.tw,kf.

35 mlearn*.tw,kf.

36 smartphone*.tw,kf.

37 smart-phone*.tw,kf.

38 ((mobile or cell) adj2 phone*).tw,kf.

39 iphone*.tw,kf.

40 android*.tw,kf.

41 ipad*.tw,kf.

42 Personal digital assistant*.tw,kf.

43 handheld computer*.tw,kf.

44 Mobile App?.tw,kf.

45 Mobile Application?.tw,kf.

46 webcast*.tw,kf.

47 webinar*.tw,kf.

48 flipped classroom*.tw,kf.

49 Serious game*.tw,kf.

50 Serious gaming.tw,kf.

51 Patient Simulat*.tw,kf.

52 Virtual patient*.tw,kf.

53 ((educat* or instruct* or learn* or simulat* or train* or teach* or interactiv*) adj2 technolog*).tw,kf.

54 Massive Open Online Course?.tw,kf.

55 Mooc?.tw,kf.

56 (Canvas network or Coursera or Coursesites or edx or Futurelearn or iversity or miriada x or moodle or novoed or openlearning or open2study or plato or spoc or udacity or pingpong).tw,kf.

57 or/7-56

58 6 and 57

59 Education.fs.

60 Education/

61 Teaching/

62 Learning/

63 exp Inservice Training/

64 Curriculum/

65 educat*.tw,kf.

66 learn*.tw,kf.

67 train*.tw,kf.

68 instruct*.tw,kf.

69 teach*.tw,kf.

70 or/59-69

71 Health Personnel/

72 exp Allied Health Personnel/

73 Anatomists/

74 "Coroners and Medical Examiners"/

75 exp Dental Staff/

76 exp Dentists/

77 Health Educators/

78 Infection Control Practitioners/

79 Medical Laboratory Personnel/

80 exp Medical Staff/

81 exp Nurses/

82 exp Nursing Staff/

83 Personnel, Hospital/

84 Pharmacists/

85 exp Physicians/

86 Physician*.tw,kf.

87 Doctor*.tw,kf.

88 Nurs*.tw,kf.

89 Surg*.tw,kf.

90 Health Personnel.tw,kf.

91 healthcare professional*.tw,kf.

92 radiolog*.tw,kf.

93 dentist*.tw,kf.

94 Pharmacist*.tw,kf.

95 Hospital Administrator*.tw,kf.

96 Podiatr*.tw,kf.

97 Psycholog*.tw,kf.

98 Psychiatr*.tw,kf.

99 An?esthesi*.tw,kf.

100 Clinician*.tw,kf.

101 Dermatolog*.tw,kf.

102 General practioner*.tw,kf.

103 Cardiolog*.tw,kf.

104 Oncolog*.tw,kf.

105 Rheumatolog*.tw,kf.

106 Neurolog*.tw,kf.

107 Patholog*.tw,kf.

108 P?ediatric*.tw,kf.

109 Physiotherap*.tw,kf.

110 Physical therap*.tw,kf.

111 Occupational therap*.tw,kf.

112 dieti?ian*.tw,kf.

113 Dietetic*.tw,kf.

114 midwi?e*.tw,kf.

115 nutrition*.tw,kf.

116 orthopti*.tw,kf.

117 obstetric*.tw,kf.

118 gyn?ecolog*.tw,kf.

119 orthodont*.tw,kf.

120 Urolog*.tw,kf.

121 or/71-120

122 Health Occupations/

123 exp Allied Health Occupations/

124 Biomedical Engineering/

125 Chiropractic/

126 exp Dentistry/

127 exp Evidence-Based Practice/

128 exp Evidence-Based Practice/

129 exp Nursing/

130 Dietetics/

131 Optometry/

132 Orthoptics/

133 exp Pharmacology/

134 exp Pharmacy/

135 Podiatry/

136 Psychology, Medical/

137 Serology/

138 Specialization/

139 exp Surgical Procedures, Operative/

140 exp Radiography/

141 or/122-140

142 121 or 141

143 57 and 70 and 142

144 57 and 70 and 142

145 motor skills/

146 ((psychomotor or procedural or technical) adj3 skill*).tw,kf.

147 (psychomotor adj3 performance).tw,kf.

148 or/144-147

149 6 and 148

150 58 or 143 or 149

151 limit 150 to yr="2014-Current"

152 (((comprehensive* or integrative or systematic*) adj3 (bibliographic* or review* or literature)) or (meta-analy* or metaanaly* or "research synthesis" or ((information or data) adj3 synthesis) or (data adj2 extract*))).ti,ab. or (cinahl or (cochrane adj3 trial*) or embase or medline or psyclit or (psycinfo not "psycinfo database") or pubmed or scopus or "sociological abstracts" or "web of science").ab. or ("cochrane database of systematic reviews" or evidence report technology assessment or evidence report technology assessment summary).jn. or Evidence Report: Technology Assessment*.jn. or ((review adj5 (rationale or evidence)).ti,ab. and review.pt.) or meta-analysis as topic/ or Meta-Analysis.pt.

153 151 and 152
