# Supplementary material for: Digital Education for Health Professionals: An Evidence Map, Conceptual Framework, and Research Agenda
Source: J Med Internet Res. 2022 Mar 17;24(3):e31977. doi: 10.2196/31977 (PMC8972116; doi:10.2196/31977)
Supplement: Multimedia Appendix 2 [file jmir_v24i3e31977_app2.docx]

##### Appendix 2. Educational outcomes reported in the included systematic reviews and their definitions

- Knowledge: learners’ factual or conceptual understanding
- Skills: learners’ ability to demonstrate a procedure or technique in an educational setting
- Attitudes toward the digital education intervention: the tendency to respond positively or negatively towards the intervention
- Satisfaction: the level of approval when comparing perceived performance in digital health education with one’s expectations
- Behavior: behaviors of learners in their clinical practice
- Patient outcome: the impact of the intervention on patients
