# Supplementary material for: Digital Education for Health Professionals: An Evidence Map, Conceptual Framework, and Research Agenda
Source: J Med Internet Res. 2022 Mar 17;24(3):e31977. doi: 10.2196/31977 (PMC8972116; doi:10.2196/31977)
Supplement: Multimedia Appendix 3 [file jmir_v24i3e31977_app3.docx]

Appendix 3. Characteristics of the included systematic reviews

| **Study ID (year, first Author, country)** | **Study objective** | **Study designs included** | **Topic** | **Participants (N)** | **Intervention** | **Comparison** | **Outcomes (Learners, Patients, Institution)** | **Evidence – GRADE assessment or other scales** | **Setting** | **Framework component** |
| --- | --- | --- | --- | --- | --- | --- | --- | --- | --- | --- |
| 2014, George  Singapore [12] | To evaluate the effectiveness of online learning on students’ knowledge, skills, attitudes and satisfaction | Randomized controlled trials | Health professions education (Medicine, nursing, physical therapy, pharmacy, dentistry) | Pre-service: Undergraduate medical, nursing, physical therapy, pharmacy or dentistry students  N=6750 | Non-simulation – online or online blended digital education | Traditional education or no intervention | Learners: Online digital education is more effective than traditional learning in knowledge gain and skill acquisition. Results on satisfaction for online digital education was mixed. There was no difference in student attitudes towards online digital education.  Institution: Some studies mentioned financial and resource-related elements of digital education, but these were not clearly reported. | Risk of bias: 31 (51%) studies with high overall ROB; 29 (47%) studies with unclear ROB in ≥ 1 category; 1 (2%) study with low overall ROB | HIC: 51  MIC: 7 | EDN – Dsg, Cnt; LRN |
| 2014, Lahti  Finland [109] | To assess the impact of digital education on nurses’ and nursing students’ knowledge, skills and satisfaction | Randomized controlled trials | Nursing | Pre-service: Nursing students;  In-service: Nurses  N=2491 | Non-simulation – online or offline digital education | Traditional education | Learners: Some improvement in knowledge and skills level, but was not statistically significant. Participants were generally satisfied with the intervention methods. | Risk of bias: All studies had high ROB in blinding (performance and detection bias) | HIC: 10  MIC: 1 | EDN – Dsg, Cnt; LRN |
| 2014, Mickan  United Kingdom [76] | To assess whether the use of handheld computers improved access to information and support clinical decision making at the point of care | Randomized controlled trials | Clinical diagnosis and decision making | Pre-service: Nursing students;  In-service: Medical and nursing staff  N=226 | Non-simulation; mLearning (offline, with preloaded guides or software) | Traditional education or other forms of mLearning (without preloaded guides or software) | Learners: Improved clinical knowledge, adherence to guidelines, clinical and diagnostic decision making.  Patients: Not reported. | Risk of bias: One study with low ROB for all categories, six studies with unclear ROB ≥ 2 categories, five studies with high ROB ≥ 1 category | HIC: 7 | EDN – Dsg, Cnt; LRN |
| 2014, Qiao  China [107] | To determine the effectiveness of virtual simulator training to teach novices in gastrointestinal endoscopy | Randomized controlled trials | Gastrointestinal endoscopy (colonoscopy, gastroscopy) | In-service: Internal medicine and general surgical novices  N=354 | Simulation – digital psychomotor skills trainer (virtual reality) | No simulation | Learners: Simulator training improved independent procedure completion for gastroscopy, but had no significant impact on independent procedure completion and total procedure time in colonoscopy.  Patients: Simulation training may contribute to increased patient comfort. | GRADE: Low-quality evidence;  Risk of bias: Four studies rated as high ROB due to blinding; two studies rated as high ROB for allocation sequence concealment | HIC: 22 | EDN – Dsg, Cnt; LRN |
| 2014, Rasmussen  United Kingdom [74] | To compare the effectiveness of offline digital education on students’ knowledge, skills, satisfaction and attitudes towards digital education | Randomized controlled trials | Health professions education (Medicine, nursing, dentistry) | Pre-service: Undergraduate medical, nursing or dentistry students  N=4955 | Non-simulation – offline or offline blended digital education | Traditional education | Learners: Majority of studies showed that offline digital education was equivalent or superior to traditional learning. | Risk of bias: Majority of studies were considered of low quality because of high ROB. Majority of studies had unclear ROB in ≥ 1 category | HIC: 42  MIC: 5 | EDN – Dsg, Cnt; LRN |
| 2014, Thepwongsa  Australia [75] | To assess the effectiveness of online continuing medical education for general practitioners | Randomized or non-randomized controlled trials, quasi-experimental studies | Medical education (continuing) | In-service: General practitioners, other healthcare professionals  N=2874 | Non-simulation – online digital education | Traditional education | Learners: Positive learning outcomes were reported on knowledge, but outcomes on satisfaction were not reported. Changes in clinical behaviour were mixed.  Patients: Insufficient evidence to conclude. | Quality assessment according to Jadad scale[119]: Three studies scored 3, two studies scored 2, one study scored 1, five studies scored 0 | HIC: 14 | EDN – Dsg, Cnt; LRN |
| 2015, Cheng  Canada [47] | To compare the use of low versus high fidelity mannequins in advanced life support training | Randomized or non-randomized controlled trials | Advanced life support | Pre-service: Medical students, nursing students;  In-service: Residents, allied health professionals, neonatal fellows  N=1162 | Simulation – digital psychomotor skills trainer (high fidelity mannequins | Low fidelity mannequins | Learners: High fidelity mannequins showed no benefit for knowledge gain at course conclusion. There was also no benefit for skill performance between course conclusion and one year, and at one year. | GRADE: Knowledge outcomes from RCT – low quality evidence; knowledge outcomes from non-RCT – very low quality evidence; skills performance (1 year) – low quality evidence; skill performance (at course conclusion and between 1 year) – very low quality evidence | HIC: 14 | EDN – Dsg, Cnt; LRN |
| 2015, Jayakumar  United Kingdom [41] | To assess the effectiveness and relevance of digital education in surgical education | Pre/post studies with no comparison groups, randomized controlled trials | Surgery | Pre-service: Medical and dental students;  In-service: General surgery residents, surgeons, residents, oral health specialists  N=5222 | Simulation – virtual patients (standalone or blended with traditional);  Non-simulation – online digital education | Traditional learning, other digital education modalities | Learners: Mix of improvement or no difference in knowledge scores were reported between intervention and control groups. Long-term retention appeared to be improved with spaced delivery. Result for satisfaction was not reported. Significant improvement in skills in the intervention group, but was unclear if it translates to technical proficiency. | Not reported | HIC: 35  MIC: 2  LIC: 1 | EDN – Dsg, Cnt, Pdg; LRN |
| 2015, Osborne  Australia [87] | To evaluate the effectiveness of high-fidelity simulators to teach obstetric ultrasound skills to health professionals | Case-controlled studies, case study, comparative study with no control | Obstetric ultrasound skills | Pre-service: Medical students;  In-service: Obstetricians, certified obstetricians and gynaecologists  N=91 | Simulation – digital psychomotor skills trainer (high fidelity ultrasound simulators) | Theoretical training | Learners: Improved obstetric ultrasound skills. Two studies demonstrated that skills developed using simulators improved clinical performance. | McMaster University Critical Review for Quantitative Studies: Consistent sources of potential bias due to contamination, co-intervention and lack of sample size justification | HIC: 4 | EDN – Dsg, Cnt; LRN |
| 2015, Piromchai  Australia [77] | To assess the effectiveness of virtual reality simulation-based surgical training to achieve surgical outcomes | Randomized controlled trials, controlled trials | Surgery (of ear, nose, or throat) | Pre-service: Medical students;  In-service: Otolaryngology residents, ophthalmology residents, surgical trainees  N=210 | Simulation – digital psychomotor skills trainer (virtual reality) | Traditional education | Learners: Trainees/students who received virtual reality simulation training exhibited better psychomotor scores, procedural scores, endo-product scores, anatomical identification (1 study) scores and completed surgical task in less time | GRADE: Low quality evidence for procedural score; very low quality evidence for psychomotor score | HIC: 9 | EDN – Dsg, Cnt; LRN |
| 2015, Kato  Japan [114] | To assess the effectiveness of high fidelity simulators in emergency training in obstetrics | Randomized controlled trials | Acute obstetric emergencies | In-service: Obstetrician, gynaecologists, nurses, midwives  N=257 | Simulation – digital psychomotor skills training (high fidelity mannequin-based simulator) | Low fidelity simulation, lectures or no training | Learners: Knowledge outcomes were mixed (one study measured increased knowledge score, one study found no significant difference). Majority of studies reported improved performance in obstetric skills following simulation training. | Risk of bias: All studies had unclear ROB in ≥ 2 categories. One study had high ROB in 1 category | HIC: 5 | EDN – Dsg, Cnt; LRN |
| 2016, Aim  France [78] | To determine the effectiveness of using virtual reality in orthopaedic surgical training | Randomized controlled trials, observation studies | Orthopaedic surgery | Pre-service: Medical, nursing or paramedical students;  In-service: Surgery residents, novice or experience surgeons  N=303 | Simulation – digital psychomotor skills trainer (virtual reality) | Textbook-based, institution-specific orthopaedic education and training | Learners: Overall performance in skills outcomes (e.g., time to complete tasks, path length). Satisfaction was not reported.  Patients: Assessed in real patients in one study, but no outcomes reported. | Not reported | HIC: 9 | EDN – Dsg, Cnt; LRN |
| 2016, Alaker  United Kingdom [66] | To evaluate the impact of virtual reality simulation training in laparoscopic abdominal surgery | Randomized controlled trials | Laparoscopic surgery | Pre-service: Medical students;  In-service: Novice or experience laparoscopic surgeons  N=1295 | Simulation – digital psychomotor skills trainer (virtual reality) | No training, video trainer or box trainer | Learners: Overall improvement in skills performance in virtual reality trained group, compared to no training, video trainers or box trainers | Risk of bias: All studies with high ROB in ≥ 1 category | HIC: 31 | EDN – Dsg, Cnt; LRN |
| 2016, Boutefnouchet  United Kingdom [44] | To assess the effectiveness of arthroscopic simulation training on surgical skills in the operating room | Randomized controlled trials | Arthroscopic surgery | In-service: Orthopedic or speciality trainees, surgical residents  N=39 | Simulation – digital psychomotor skills trainer (computer-assisted) | Standard case-based training under the supervision of more senior surgeons | Learners: Simulator training improved performance in the operating theatre as well as skills retention. | Not reported | HIC: 2 | EDN – Dsg, Cnt; LRN |
| 2016, den Harder  The Netherlands [88] | To evaluate the effects of radiology digital education programs with and without image interaction on learning outcomes | Single group cross-sectional or post-test studies, single group pre/post studies, randomized or non-randomized controlled trials | Radiology and anatomy | Pre-service: Undergraduate medical students  N=incomplete information; 3852 participants reported from 17/19 studies, 2/19 studies did not provide participant number | Simulation – virtual reality with three-dimensional images | Slide projectors, two-dimensional or static images | Learners: Radiology skills and knowledge improved in general after digital education; knowledge and x-ray image interpretation skills also improved. | MERSQI: Score was variable, with range 7-14.5 (out of 18) | HIC: 18  One study was conducted worldwide | EDN – Dsg, Cnt; LRN |
| 2016, Glassman  United Kingdom [89] | To evaluate the effects of video game use on laparoscopic surgical skills performance | Randomized controlled trials | Laparoscopic surgical skills | Pre-service: Medical students;  In-service: Surgical residents, laparoscopic experts (surgeons, residents gynaecologists who performed > 30 laparoscopic procedures in last 3 years)  N=142 | Simulation – video games | No video games | Learners: Limited evidence to show that the use of video games improved surgical skills. Three studies showed a reduction in error or time to perform tasks after video game use, while the other two studies showed no difference between the control and intervention group. | MERSQI: All studies were assessed to be 14 or over (maximum score of 18) | HIC: 5 | EDN – Dsg, Cnt; LRN |
| 2016, Liu  China [60] | To evaluate the effectiveness of digital blended learning for health professionals | Pre/post or post-test studies only, randomized or non-randomized controlled trials | Health professions education | Pre-service: Medical or nursing students;  In-service: Nurses, physicians, public health workers  N=2238 | Non-simulation – online or offline blended digital education | Face-to-face lectures, classroom teaching | Learners: Overall positive impact on knowledge when comparing blended with no intervention or non-blended interventions. | GRADE: Low (blended vs no intervention) or moderate (blended vs non-blended intervention) quality evidence for knowledge scores | Developed countries: 44  Developing countries: 12 | EDN – Dsg, Cnt; LRN |
| 2016, Maertens  Belgium [90] | To evaluate the effectiveness of digital modalities as a teaching tool for surgical training compared with | Randomized controlled trials | Surgical skills in urology, trauma/critical care, otolaryngology, OBGYN, ophthalmology | Pre-service: Medical students;  In-service: Junior surgical trainees, senior trainees and/or fellows, trained surgeons, nurses  N=7871 | Non-simulation – online or offline digital education (internet or software-based platforms);  Simulation – virtual reality, virtual patient, serious gaming | Traditional education or no intervention | Learners: Majority of studies showed either greater or similar effectiveness when compared with non-digital interventions or no interventions. Only two studies demonstrated skills performance in the clinical environment.  Patients: There were no studies that assessed changes in patient outcomes. | GRADE: Majority of studies were moderate to high quality. The rest of the studies scored low or very low (no specific information on the outcome measures) | Information not available | EDN – Dsg, Cnt; LRN |
| 2016, Mok  Hong Kong [48] | To assess the effectiveness of high fidelity patient simulation to teach clinical reasoning skills | Randomized or quasi-randomized controlled trials | Clinical reasoning | Pre-service: Nursing students  N=859 | Simulation – digital psychomotor skills trainer (high fidelity patient simulator) | Traditional education, low- to medium-fidelity simulation or online discussion | Learners: Lack of support that high-fidelity patient simulation was more effective than other teaching modalities to teach clinical reasoning, but it is at least as effective. No significant difference in self-efficacy and satisfaction scores (each was reported in one study) | Quality assessment (Jadad scale[119]): Overall score of 1 or 2, out of maximum score of 7 | HIC: 10  MIC: 1 | EDN – Dsg, Cnt; LRN |
| 2016, Santos  Brazil [49] | To determine the effectiveness of digital education in oral radiology education | Randomized or non-randomized controlled trials | Oral radiology | Pre-service: Dental students  N=964 | Non-simulation – online or offline digital education | Traditional education | Learners: Impact on knowledge gain was variable, with 6/11 studies showing no significant improvement, while 2/11 showed a significant gain in knowledge using digital education. Majority of studies included reported positive attitude towards digital education in oral radiology. Performance on clinical procedures was inconclusive. | MINORS criteria: 4 of 6 non-randomised controlled trials scored poorly;  Risk of bias: randomized controlled trials had overall high or unclear ROB | HIC: 7  MIC: 4 | EDN – Dsg, Cnt; LRN |
| 2016, Sinclair  Australia [91] | To evaluate the effectiveness of digital education programs on health care professional behaviour and patient outcomes | Randomized controlled trials | Health professions education (Physical therapy, sleep medicine, operation management, emergency preparedness, pediatric prescription, surgery) | Pre-service: Medical, nursing or physical therapy students;  In-service: Nurses, doctors  N=638 | Non-simulation – online digital education | Traditional education or no intervention | Learners: Digital education was at least equivalent to traditional learning approaches, or superior to no intervention on health care professional behaviour.  Patients: There were no studies that reported an impact on patient outcomes. | JBI-MAStARI: Methodological quality reported was variable, score range 5 to 8 of maximum 10. One study scored 5, three studies scored 6, one study scored 7, two studies scored 8 | HIC: 6  MIC: 1 | EDN – Dsg, Cnt; LRN |
| 2016, Tarpada  United States [113] | To evaluate the potential for digital education in orthopaedic education | Randomized controlled trials and pilot studies | Orthopaedic surgery | Pre-service: Medical students;  In-service: Orthopedic surgery residents, surgery residents  N=incomplete information; 1596 participants from 8/9 studies | Non-simulation – online or offline digital education (with blended option);  Simulation – virtual patient (only 1 study without control) | Traditional education, cadaver-based training | Learners: Studies showed general improvement in OSCE post-test scores or multiple-choice exam results. | Not reported | HIC: 7  MIC: 1  One study undisclosed | EDN – Dsg, Cnt; LRN |
| 2017, Lui  Canada [92] | To examine temporal bone surgical performance following the use of virtual reality temporal bone simulation | Cohort studies, randomized controlled trials | Temporal bone surgery | Pre-service: Medical students;  In-service: Medical residents  N=179 | Simulation – digital psychomotor skills trainer (virtual reality) | Cadaver-based training | Learners: Statistically significant improvement in performance, but improvement of performance in the operating room was unclear. | Not reported | HIC: 8 | EDN – Dsg, Cnt; LRN |
| 2017, Pfandler  Germany [79] | To assess the quality of virtual reality-based training in spinal surgery | Pre/post-test studies without a control group, cross-sectional studies, cohort studies | Spine surgery | Pre-service: Medical students;  In-service: Surgeons, orthopaedic residents/house officers, surgical residents  N=1089 | Simulation – digital psychomotor skills trainer (virtual reality) | Traditional education using models | Learners: Simulator training was effective and outperformed the non-simulator trained group. Knowledge and skills improved. Behaviour was not fully addressed.  Patients: Proxy measures for patient care quality were reported – less tissue damage. | MERSQI: Considerable variation in score; average total MERSQI = 11.47 out of 18, SD = 1.81, range = 7.0-13.5;  Overall quality was low to medium | HIC: 19 | EDN – Dsg, Cnt, Evl; LRN |
| 2017, Richmond  United Kingdom [103] | To review the effectiveness of online education methods to train clinicians’ knowledge and practical skills | Randomized controlled trials | Health professions education (mix of theoretical knowledge, applied knowledge/skills or complex interventions) | In-service: Medical residents, physicians, nurses, mental health psychologist or counsellors, allied health professionals  N=3825 | Non-simulation – online digital education | Traditional education | Learners: There was little difference in knowledge and clinical behaviour between online digital education and alternative training, and too few studies to provide a conclusion on the effects of online training for practical skills, self-efficacy and satisfaction.  Patients: There were no studies that reported patient outcomes. | GRADE: Low-quality evidence (online vs interactive workshop for clinical behaviour, or workshop and lecture for knowledge); very low-quality evidence (online training vs manual for knowledge) | HIC: 13  MIC: 1 | EDN – Dsg, Cnt; LRN |
| 2017, Rohwer  South Africa [93] | To assess the effectiveness of online and offline digital education to increase evidence-based health care competencies | Randomized or non-randomized controlled trials | Evidence-based health care | Pre-service: Medical and nursing students;  In-service: Medical doctors, nurses, physiotherapists, physician assistants, athletic trainers  N=3825 | Non-simulation – online and offline digital education or blended learning | No intervention or traditional education | Learners: The use of digital education and blended learning, compared to no learning, improved knowledge, skills and attitudes. No difference was observed when compared digital education with face-to-face learning. | Risk of bias: Overall moderate risk of bias | HIC: 22  MIC: 2 | EDN – Dsg, Cnt; LRN |
| 2017, Tomesko  United States [50] | To determine the effectiveness of computer-assisted instruction to teach physical examination skills | Randomized controlled trials | Physical examination | Pre-service: Medical students;  In-service: Physical therapists, orthodontic graduate students  N=436 | Non-simulation – online or offline computer-assisted instruction | Traditional education, laboratory sessions | Learners: Effectiveness from intervention was unclear as outcomes showed either increased effectiveness, no difference or less effective than control | GRADE: Overall low quality evidence | HIC: 7 | EDN – Dsg, Cnt; LRN |
| 2017, Voutilainen  Finland [105] | To determine the effectiveness of digital education on nursing education learning outcomes | Randomized controlled trials, controlled trials, quasi-experimental studies, historical comparison | Nursing education | Pre-service: Nursing students  N=1425 | Non-simulation – online or offline digital education, mLearning | Traditional education or no additional training | Learners: Digital education improved knowledge or skill scores more than traditional learning. The range of improvement was very wide. | Hawker et al appraisal criteria: 9/10 studies evaluated as good quality, but ROB still existed.  Risk of bias: four studies with high ROB in 1 category; two studies with high ROB in 2 categories; three studies with high ROB in 3 categories | HIC: 10 | EDN – Dsg, Cnt; LRN |
| 2018, Ahmet  Turkey [51] | To evaluate the use of video-based surgical education to teach technical and non-technical surgical skills | Randomized controlled trials or case-controlled studies | Surgery (laparoscopic, cataract and glaucoma, dental, urology-obstetrics and gynaecology, general) | Pre-service: Medical students;  In-service: Residents or staff surgeons, intern physicians  N=507 | Non-simulation – online or offline digital education | Conventional master-apprentice model, or three-dimension animations with video sequences, simulator exercises with video | Learners: Significant improvement when compared with traditional education. The use of video feedback in simulator exercises improved technical skills | NIH Study Quality Assessment Tool: Fair to good quality. Scores ranged from 4 to 13 (one study scored, 4, three studies scored 7, three studies scored 8, one study scored 12, one study scored 13) | HIC: 8  MIC: 1 | EDN – Dsg, Cnt; LRN |
| 2018, Gorbanev  Colombia [112] | To determine the pedagogical strategies and the effectiveness of serious games | Randomized or non-randomized controlled trials, single group cross-sectional or post-test only study, single group pre/post-test study | Medical education | Pre-service: Medical students;  In-service: Residents  N=1872 | Simulation – serious games | No serious games | Learners: All articles reported positive effects of games on learning (knowledge and skills) and students’ motivation. However, the degree of these effects and the level of evidence varied.  Patients: No studies reported patient outcomes. | MERSQI: Overall quality of evidence was moderate; articles averaged 10.8 points out of a total of 18. Range 4.5-15.5 | HIC: 20  MIC: 1 | EDN – Dsg, Cnt; LRN |
| 2018, Kang  Republic of Korea [80] | To evaluate the effectiveness of web-based nursing programs for nurses and nursing students | Randomized or quasi-randomized controlled trials | Nursing education | Pre-service: Nursing students;  In-service: Nurses  N=1056 | Non-simulation – online or online blended digital education | Traditional practice or clinical practicum | Learners: Online blended learning had a positive effect on the knowledge level and clinical performance with significant effect size difference observed; web-based programmes also had a positive effect on the knowledge level and clinical performance, with a significant difference in effect size when participants were nursing students. Self-efficacy/self-directed learning ability and self-confidence were not well-reported | Risk of bias: All articles had high ROB in ≥ 1 category; seven articles had unclear ROB in one other category | HIC: 8  MIC: 3 | EDN – Dsg, Cnt; LRN |
| 2018, Khan  Canada [67] | To determine the effectiveness of virtual reality simulation training to supplement and/or replace early conventional endoscopy training | Randomized controlled trials | Gastrointestinal endoscopy | In-service: Medical residents or fellows, surgical trainees or residents, physicians, nurses  N=421 | Simulation – digital psychomotor skills trainer (virtual reality) | Conventional patient-based endoscopy training (apprenticeship model) or other methods of simulation | Learners: When compared with no training, there was an improvement in independent procedure completion and overall global rating of performance or competency, but no significant difference in performance time, complication occurrence or patient discomfort. When compared with patient-based or other forms of training, no difference in outcomes reported. | GRADE: Very low-quality evidence for overall performance, competence, and patient discomfort; low-quality evidence for competency; moderate-quality evidence for independent procedure completion and completion occurrence | HIC: 26 | EDN – Dsg, Cnt; LRN |
| 2018, Koohestani  Iran [68] | To evaluate the educational effects of mobile learning for medical sciences students | Pre/post-test or post-test only studies with or without control groups | Health professions education | Pre-service: Medical, nursing, dentistry or physiotherapy students;  In-service: Residents  N=1063 | Non-simulation – mobile learning | Traditional education or no intervention | Learners: Improvement in learners’ clinical performance, theoretical knowledge, attitudes/perceptions and satisfaction towards mobile learning. There was also improvement in their prescription using the drug calculator. | BEME Review of Education Portfolio: 11 high-quality papers, 10 medium quality papers, no low-quality papers (assessed based on 11 quality indicators) | HIC: 15  MIC: 6 | EDN – Dsg, Cnt; LRN |
| 2018, Lee  Republic of Korea [81] | To evaluate the effectiveness of using mobile technology in undergraduate nursing education | Randomized controlled trials, quasi-experimental studies | Nursing education | Pre-service: Undergraduate nursing students  N=909 | Non-simulation – mobile learning | Traditional lectures, other digital education modalities (web-based, high fidelity patient simulators) | Learners: Majority of studies did not report improvement in the areas assessed. Some studies reported smartphone-based applications could promote learning motivation but not improve clinical skills and knowledge. | JBI-MAStARI:  RCT – weak quality due to high ROB (range of scores from 5 to 10 of 13);  Quasi – high quality due to low ROB (range of scores from 7 to 9 of 9) | HIC: 14 | EDN – Dsg, Cnt; LRN |
| 2018, Liossi  United Kingdom [52] | To evaluate the effectiveness of online educational resources for educating health professionals in pain assessment and management | Randomized or non-randomized controlled trials, single group pre/post-test studies | Pain assessment and management | Pre-service: Medical, nursing or dental students;  In-service: Surgeons, residents nurses, postgraduate trainees, physiotherapists, psychologists  N=incomplete information: 4207 participants from 28/32 studies; 4/32 studies reported involving staff from medical centres or nursing homes | Non-simulation – online digital education | Traditional education or no intervention | Learners: Knowledge and skills towards pain management improved, but there were no significant differences in confidence and attitudes.  Patients: Insufficient information on patient outcomes. | Risk of bias: 9/17 RCTs have high ROB for random sequence generation; 13/17 studies have high or unclear ROB for allocation concealment | HIC: 31  MIC: 1 | EDN – Dsg, Cnt; LRN |
| 2018, Rambarat  United States [69] | To appraise the current literature on the use of simulation to teach echocardiography across all specialities | Randomized controlled trials, pre/post comparison studies, cohort studies | Echocardiography in cardiology, anesthesiology, emergency medicine and pulmonary critical care | Pre-service: Medical students;  In-service: Graduate medical education, continuing medical education  N=503 | Simulation – digital psychomotor skills trainer (high fidelity mannequin simulators) | Traditional education | Learners: Improvement in anatomical identification, image quality and acquisition time and identification of structures when outcomes were measured on live patients.  Patients: Clinical impact in terms of identification of new clinical diagnoses via the use of point-of-care echo intraoperatively.  Institution: Limited information on change in institutional practice. | BEME appraisal:  Rating of 3 – 11 studies;  Rating of 4 – 8 studies;  Rating of 1 – 3 studies;  Rating of 2 – 2 studies | HIC: 21  Three studies undisclosed | EDN – Dsg, Cnt, Evl; LRN |
| 2018, Vaona  Italy [70] | To assess the effectiveness of digital education programs to improve licensed health professionals’ knowledge, skills and behaviours, and to improve patient outcomes | Randomized controlled trials | Health professions education | In-service: Nurses, paediatricians, physicians, physiotherapists, childcare health consultants  N=5679 | Non-simulation – online or offline digital education | Traditional education | Learners: Little or no difference in health professionals’ behaviour and knowledge. Effect on skills is unclear.  Patients: Digital education provides little or no difference in patient outcomes. | GRADE: Very low-quality evidence for health professionals’’ skills; low-quality evidence for behaviour, knowledge and patient outcomes | HIC: 14  MIC: 2 | EDN – Dsg, Cnt; LRN |
| 2018, Zhou  China [115] | To evaluate the effectiveness of mobile learning on medical and nursing education | Randomized controlled trials | Health professions education | Pre-service: Medical or nursing students;  In-service: Medical residents, nurses  N=844 | Non-simulation – mobile learning | Traditional education or no intervention | Learners: Mobile learning had mixed outcomes on knowledge and performance of participants, showing either improvements in clinical knowledge and performance or showing no significant difference compared to traditional education or no intervention. | Risk of bias: All studies had high ROB in ≥ 1 category. Nine studies had unclear ROB in ≥ 1 category | HIC: 10  MIC: 1 | EDN – Dsg, Cnt; LRN |
| 2019, Botelho  Hong Kong [82] | To evaluate the effectiveness of digital education or blended learning in undergraduate dental radiology | Cohort studies, single group pre/post-test studies | Dental radiology | Pre-service: Dentistry students  N=1039 participants from 15/17 studies | Non-simulation – online or online blended digital education | Other online digital education modalities | Learners: Positive outcomes related to knowledge and performance. Students’ attitudes towards online or blended digital education were positive. | Not reported | HIC: 13  MIC: 4 | EDN – Dsg, Cnt; LRN |
| 2019, Brusamento  United Kingdom [59] | To evaluate the effectiveness of different digital education modalities to impact paediatric health professionals | Randomized controlled trials | Pediatric care | In-service: Pediatricians, nurses, midwives and health extension works, childcare health consultants, emergency medicine health workers, doctors  N=1382 | Non-simulation – online or offline digital education, blended offline digital education;  Simulation – digital psychomotor skills trainer (high fidelity), virtual reality environment | Traditional education or low fidelity mannequin | Learners: Digital education was either as effective or more effective than control interventions. High fidelity mannequins were more effective at improving psychomotor skills than low fidelity mannequins.  Patients: Only one study assessed patient outcome, which found no difference between interventions and the control group. | GRADE: Overall low quality of evidence for skills, knowledge and satisfaction outcomes | HIC: 17  MIC: 2  LIC: 1 | EDN – Dsg, Cnt; LRN |
| 2019, Campbell  United Kingdom [94] | To analyze the effectiveness of online cancer education for nurses and allied health professionals | Randomized controlled trials, cohort studies, qualitative studies | Cancer education | In-service: Nurses and allied health professionals  N=2815 participants from 28/30 studies. Two studies did not report number of participants. | Non-simulation – online or blended online digital education | Traditional education | Learners: There is no evidence to suggest that online delivery of education is better or worse in education terms than face-to-face delivery.  Patients: Online delivery does not hinder the achievement of clinical outcomes. | Not reported | HIC: 28  MIC: 2 | EDN – Dsg, Cnt, Evl; LRN |
| 2019, Divakar  Singapore [97] | To evaluate the effectiveness of digital education on health professionals’ management of domestic violence | Randomized controlled trials | Domestic violence management | Pre-service: Dental students;  In-service: Dentists, physicians in emergency medicine, orthopaedics, obstetrics and gynaecology, nurses  N=631 | Non-simulation – online or offline digital education | Traditional education or no intervention | Learners: Digital education increased post-intervention knowledge, attitude, self-efficacy and skills towards managing domestic violence in patients. | GRADE: Low-quality evidence for knowledge, attitude and skills; moderate-quality evidence for self-efficacy | HIC: 6 | EDN – Dsg, Cnt; LRN |
| 2019, Dunleavy  Singapore [11] | To evaluate the effectiveness of using mLearning education interventions to deliver education to pre- and in-service health professionals | Randomized controlled trials | Health professions education | Pre-service: Medical, nursing, dental or physiotherapy students;  In-service: Nurses, midwives, physicians, internal or family medicine residents, neurosurgeon trainees, health extension workers, trauma and critical care fellows  N=3175 | Non-simulation – mobile learning | Traditional education, another form of mobile learning or other forms of digital education | Learners: mLearning improved knowledge and skills when compared with traditional education. Findings for attitude and satisfaction were inconclusive. There was no change in clinical practice or behaviours.  Patients: No significant difference in patient-reported outcomes (one study). | Risk of bias: 55% of studies had a high overall risk of bias | HIC: 24  MIC: 4  LIC: 1 | EDN – Dsg, Cnt, Evl; LRN |
| 2019, Gentry  United Kingdom [14] | To evaluate the effectiveness of serious gaming and gamification education in improving health professionals’ knowledge, skills, attitudes, satisfaction and patient outcomes | Randomized controlled trials | Health professions education | Pre-service: Medical, nursing, dental, speech-language and hearing science students;  In-service: Surgical residents, urologists, anesthesiology residents, primary care physicians  N=3634 | Simulation – serious games and gamification | Traditional education, other digital education intervention or other serious gaming/ gamification intervention | Learners: For knowledge, serious gaming/gamification was superior to traditional education. Some evidence showed that skills improved. No statistically significant difference for attitude outcomes, while evidence for impact on satisfaction was mixed.  Patients: Shorter time to control blood pressure in the intervention group. However, insufficient data to conclude. | GRADE: Low-quality evidence for knowledge, skills and satisfaction; very low-quality evidence for attitude;  Risk of bias: 25 studies were considered to be at high overall ROB | HIC: 26  MIC: 4 | EDN – Dsg, Cnt, Pdg, Evl; LRN |
| 2019, George  Singapore [13] | To evaluate the effectiveness of online digital education in improving doctors’ knowledge, skills, attitude and satisfaction | Randomized controlled trials, quasi-experimental with pre/post test | Health professions education | In-service: Primary care, general or internal medicine practitioners, surgeons, paediatricians, radiation oncologists, otolaryngologists, orthopaedics, gastroenterologists, rheumatologists, anesthesiologists, occupational physicians, surgical or OBGYN residents  N=16895 | Non-simulation – online or blended online digital education | Traditional education | Learners: Online and blended online learning may be equivalent to traditional education for improving physicians’ knowledge, skills, attitude, satisfaction, practice or behaviour.  Patients: Online or blended online digital education may be as effective as traditional education in improving patient outcomes.  Institution: Three studies reported pharmaceutical cost containment in the intervention group. | GRADE: Very low-quality evidence for knowledge; low-quality evidence for skills, attitude and satisfaction | HIC: 91  MIC: 2 | EDN – Dsg, Cnt; LRN |
| 2019, Guedes  Brazil [110] | To evaluate the effectiveness of virtual reality simulator compared to box trainers to train minimally invasive surgery | Randomized controlled trials | Minimally invasive surgery | Pre-service: Medical students;  In-service: Doctors, residents of surgical specialties, surgeons  N=695 | Simulation – digital psychomotor skills trainer (virtual reality) | Box trainers | Learners: Virtual reality simulator-trained group had better operative performance, and significantly shorter time to complete a basic peg transfer. No significant difference in time to complete a minimally invasive surgery, ligation loop or series of basic tasks, performance score for basic or advanced tasks. | GRADE: Moderate quality evidence in time to complete basic task, ligation loop and all basic tasks; low quality evidence in time to complete a minimally invasive surgery, camera navigation, performance score in basic and advanced tasks; | HIC: 19  MIC: 1 | EDN – Dsg, Cnt; LRN |
| 2019, Huang  Singapore [101] | To evaluate the effect of digital education on health professionals’ knowledge, skills, attitudes, competencies and behaviours on patients’ diabetes management | Randomized controlled trials | Diabetes management | Pre-service: Pharmacy or medical students;  In-service: Internal medicine residents, primary care physicians, paediatric doctors, nurses, dieticians  N=2263 | Non-simulation – online or offline digital education;  Simulation – scenario-based simulation (simulation software), digital psychomotor skills trainer (high fidelity mannequin), serious gaming | Traditional education or other digital education methods | Learners: Digital and blended education may improve health professionals’ knowledge and skills compared with traditional education.  Patients: Little or no difference in reported patient outcomes.  Institution: Little or no difference in economic impact. | Risk of bias: Seven studies were judged at high ROB, three studies had unclear ROB for ≥ 3 categories | HIC: 10  MIC: 2 | EDN – Dsg, Cnt; LRN |
| 2019, Kim  Republic of Korea [111] | To evaluate the effects of smartphone-based mobile learning for nurses and nursing students | Randomized or non-randomized controlled trials | Nursing education | Pre-service: Nursing students  N=993 | Non-simulation – online or offline mobile learning | Traditional education | Learners: Knowledge, skill, confidence in performance, attitude, satisfaction and cognitive load were analyzed. Postintervention, there was a significant difference in knowledge, skill, attitude and confidence in performance. There was a difference in confidence in performance and cognitive load, but no significance. | Risk of bias: 41.7% of studies had high or unclear ROB in random sequence generation; 58.3% studies had high or unclear ROB in allocation concealment; 66.7% had high or unclear ROB in blinding of study participants and personnel | HIC: 9  MIC: 2 | EDN – Dsg, Cnt; LRN |
| 2019, Kononowicz  Poland [83] | To evaluate the effectiveness of virtual patient simulation to deliver pre- and in-service health professions education | Randomized controlled trials | Health professions education (Medicine, nursing, pharmacy, physical therapy, osteopathic medicine, dentistry) | Pre-service: Medical, pharmacy, physical therapy, dentistry, osteopathic medicine students;  In-service: Nurses, medical staff  N=4696 | Simulation – virtual patients | Traditional education or other forms of digital education | Learners: Virtual patient simulation is as effective as traditional education on knowledge scores. Better skills outcomes were measured (clinical reasoning, procedural skills and mix of procedural and team skills). No difference for satisfaction outcome, while there was insufficient data for attitude outcome. Professional behaviour towards real patients was rated higher. | GRADE: Very low to low-quality evidence for knowledge, skills, attitudes and satisfaction | HIC: 44  MIC: 7 | EDN – Dsg, Cnt; LRN |
| 2019, Kyaw  Singapore [32] | To evaluate the effectiveness of digital education or blended digital education to train health professionals in antibiotic management | Randomized controlled trials and crossover studies | Antibiotic management | In-service: Primary care physicians  N=1392 | Non-simulation – online or online blended digital education, mobile digital devices (mLearning) | Traditional education or usual practice | Learners: Postintervention knowledge scores improved compared to traditional education. No difference regarding the perception of the intervention. Most studies demonstrated a reduction in prescribing rates postintervention.  Patients: Outcomes reported were mixed or inconclusive (hospital admission rate or re-consultation rate).  Institution: Intervention costs were lower than for traditional education. The cost of dispensed antibiotics also showed a reduction. | Risk of bias: Overall ROB for most of the studies was high or unclear | HIC: 7  MIC: 1 | EDN – Dsg, Cnt; LRN |
| 2019, Kyaw  Singapore [99] | To evaluate the effectiveness of digital education to develop communication skills in medical students | Randomized controlled trials | Health professionals’ communications skills | Pre-service: Medical students  N=2101 | Simulation – virtual patients;  Non-simulation – online or online blended digital education | Traditional education or other digital education (less interactive) or no intervention | Learners: Digital education was as effective as traditional education in teaching communication skills to medical students. Online blended digital education was as effective as and more effective to teach communication skills and knowledge. Different forms of digital education did not differ in effectiveness. | GRADE: Low-quality evidence for knowledge (digital vs traditional education); low-quality evidence for knowledge, skills, attitude, satisfaction and patient-related outcomes (blended vs traditional education); very low (satisfaction), low (attitude) or moderate (skills) quality evidence (more vs less interactive digital education) | HIC: 11  MIC: 1 | EDN – Dsg, Cnt; LRN |
| 2019, Kyaw  Singapore [40] | To evaluate the effectiveness of offline digital education to deliver and improve medical students’ knowledge, skills, attitudes and satisfaction | Randomized controlled trials | Medical education | Pre-service: Medical students  N=3325 | Non-simulation – offline digital education (software programs, CD-ROMs, PowerPoint, computer-based videos) | Traditional education or different forms of offline digital education | Learners: Offline digital education is as effective, while blended offline (one study) was more effective than traditional education in improving medical students’ knowledge. Offline digital education was more effective in improving skills compared to traditional learning. The effects on attitudes and satisfaction are uncertain. | GRADE: Low-quality evidence for knowledge, skills, attitude and satisfaction.  Risk of bias: Generally unclear or high in most of the studies | HIC: 31  MIC: 5 | EDN – Dsg, Cnt; LRN |
| 2019, Kyaw  Singapore [16] | To evaluate the effectiveness of virtual reality as a training modality for health professions education to improve knowledge, cognitive skills, attitudes and satisfaction | Randomized controlled trials | Health professions education | Pre-service: Medical, nursing, dentistry or physical therapy students;  In-service: Medical residents, nurses  N=2407 | Simulation – virtual reality | Traditional education, other digital education or other virtual reality modalities | Learners: Virtual reality was more effective than traditional education, and more interactive virtual reality modality was effective than a less interactive modality, at improving knowledge and cognitive skill scores. There were no differences in attitude and satisfaction scores.  Institution (cost outcomes): A cost analysis showed that a virtual hospital-based approach increased development costs but provided increased value during implementation by reducing staff time needed for practising wayfinding skills. | GRADE: Low-quality evidence for satisfaction scores; moderate-quality evidence for knowledge, skill and attitude scores;  Risk of bias: Overall, studies were judged as having unclear or high ROB (majority of studies had unclear ROB ≥ 2 categories) | HIC only | EDN – Dsg, Cnt, Evl; LRN |
| 2019, La Cerra  Italy [84] | To analyze the effectiveness of using high fidelity patient simulation to train nursing students and nurses in the management of life-threatening clinical conditions | Randomized controlled trials, quasi-experimental studies, pseudo-randomised studies | Cardiocirculatory or respiratory scenarios | Pre-service: Nursing students  N=3382 | Simulation – digital psychomotor skills trainer (high fidelity patient simulator) | Traditional education, low fidelity mannequin, no intervention | Learners: High fidelity patient simulator showed a significant effect on knowledge and performance compared to other teaching methods. No significant differences were detected for satisfaction, self-confidence and self-efficacy. | Risk of bias: Significant publication bias detected | HIC: 31  MIC: 2 | EDN – Dsg, Cnt; LRN |
| 2019, Li  China [120] | To investigate the effects of blended learning on nursing students’ knowledge, skills and satisfaction compared to traditional education methods | Randomized or non-randomized controlled trials | Nursing education | Pre-service: Nursing students  N=574 | Non-simulation – online blended digital education | Traditional education | Learners: Significant effects on nursing students’ knowledge. Some improvement in skills compared with traditional learning. Blended learning effectively improved nursing students’ satisfaction. | Risk of bias: All studies had unclear ROB ≥ 2 categories; one study had high ROB in 1 category | HIC: 5  MIC: 3 | EDN – Dsg, Cnt; LRN |
| 2019, Nagendrababu  Malaysia [85] | To evaluate the impact of technology-enhanced learning on education outcomes in endodontics | Randomized controlled trials | Endodontic education | Pre-service: Dental students;  In-service: Endodontic postgraduate trainees  N=596 | Non-simulation – online or offline digital education;  Simulation – digital psychomotor skills trainer (virtual reality) | Traditional education | Learners: Majority of studies showed no difference in knowledge gained post-intervention and reported either no difference or varying preferences towards technology-enhanced learning. There was no difference in performance post-intervention. | Risk of bias: Most studies reported unclear overall and randomization bias, with a low risk of bias in other categories | HIC: 6  MIC: 7 | EDN – Dsg, Cnt; LRN |
| 2019, Pei  China [42] | To assess the effectiveness of online learning versus offline learning for undergraduate medical education knowledge and skills | Randomised or non-randomised controlled trials, observational quasi-experimental studies | Medical education | Pre-service: Medical students  N=1642 | Non-simulation – online and offline digital education | Offline digital education | Learners: Significant difference in post-test scores, favouring online digital education. When comparing pre/post-test scores, while no significant difference, the result trended towards online digital education. Online digital education was also more effective than offline digital education when retention test scores were compared. | MERSQI: Mean ± SD = 13.5±1.1;  Risk of bias: High risk of random sequence generation and attrition bias; unclear risk of allocation concealment, performance, reporting bias | HIC: 4  MIC: 5 | EDN – Dsg, Cnt; LRN |
| 2019, Posadzki  Singapore [15] | To evaluate the effectiveness of offline digital education in delivering health education training to health professionals | Randomized controlled trials | Health professions education | In-service: Nurses, pharmacists, mental health therapists, dentists, midwives, physical therapists, patient care personnel  N=4618 | Non-simulation – offline or offline blended digital education | No intervention, blended or traditional education | Learners: Offline digital education improved knowledge and skills post-intervention compared to traditional education, but this was dependent on the modality (e.g. CD-ROM, computer-assisted instruction or software/PowerPoint). Impact on attitude and satisfaction were uncertain.  Patients: Insufficient data on the impact on patient outcomes | GRADE: Low (knowledge) quality evidence (CD-ROM vs no or traditional education); very low (knowledge) or low (satisfaction) quality evidence (computer-assisted training vs no or traditional education); low (knowledge) quality evidence (offline vs traditional or blended education) | HIC: 19  MIC: 7 | EDN – Dsg, Cnt; LRN |
| 2019, Semwal  Singapore [86] | To evaluate the effectiveness of digital education to improve health professionals knowledge, skills, attitudes and satisfaction towards delivering smoking cessation therapy | Randomized controlled trials | Smoking cessation therapy | Pre-service: Medical or pharmacy students;  In-service: Doctors, nurses  N=2684 | Non-simulation – online or online blended, offline or offline blended digital education | Traditional education or other digital education | Learners: Knowledge gain was similar when comparing digital with traditional education, but showed greater improvement in skills when using blended education. There was inconclusive data about attitude and satisfaction. | Risk of bias: Three studies had high ROB ≥ 1 category; all other studies had unclear ROB | HIC: 10  MIC: 1 | EDN – Dsg, Cnt; LRN |
| 2019, Tudor Car  Singapore [53] | To assess the effectiveness of digital education to encourage health professionals to adopt clinical practice guidelines | Randomized controlled trials | Clinical practice guidelines | Pre-service: Medical, emergency medicine or nursing students;  In-service: Primary care physicians, physician assistants, internal medicine residents, physiotherapists, nurses  N=2382 | Non-simulation – online or offline digital education | Traditional education or non-interactive digital education | Learners: Digital education had a moderate statistically significant and non-significant impact on knowledge when compared to no intervention or traditional education, respectively. Participants were more satisfied with digital education, and there was no change in behaviour.  Patients: No impact on patient outcomes. | GRADE: Low (behavioural change, skills) or moderate (knowledge) quality evidence (digital education vs no intervention); low (skills, satisfaction, patient outcomes) or moderate (knowledge, behavioural change) quality evidence (digital vs traditional education); low (knowledge, patient outcomes) or moderate (behavioural change) quality evidence (digital education with different levels of interactivity) | HIC: 16  MIC: 1 | EDN – Dsg, Cnt, Evl; LRN |
| 2019, Tudor Car  Singapore [34] | To evaluate the effectiveness of digital problem based learning in improving health professionals’ knowledge, skills, attitudes and satisfaction | Randomized controlled trials | Medical education | Pre-service: Medical students  N=890 | Non-simulation – online or offline digital education;  Simulation – virtual reality, virtual patient | Traditional modalities in problem-based learning or traditional education | Learners: Digital problem-based learning moderately improved knowledge scores, and slightly improved post-intervention skill scores. For satisfaction and attitude, there were mixed findings or incomplete outcomes reported. | Risk of bias: Eight studies had unclear ROB in ≥ 3 categories; one study had high ROB in 1 category. Only one study had unclear ROB in only 1 category | HIC: 7  MIC: 2 | EDN – Dsg, Cnt, Pdg; LRN |
| 2019, Viljoen  South Africa [43] | To review the efficacy of computer-assisted instruction for training medical students and residents | Randomized controlled trials, cohort studies | Electro-cardiography | Pre-service: Medical students;  In-service: Residents  N=1328 | Non-simulation – online or offline computer-assisted instruction | Traditional education | Learners: Some studies reported positive attitudes while others reported less favourable attitudes towards computer-assisted instruction. Overall, CAI was not better than face-to-face teaching for acquiring ECG competence, but the effect size was inconsistent among the studies. CAI showed no significant difference in retention of ECG competence, but this was only assessed in one study. Repeated sessions, blended learning approach and incorporating case scenarios improved ECG competence. | MERSQI: Mean±SD is 12.73±1.76 (moderate to high);  Risk of bias: Selection and/or performance bias in nine studies; three studies had attrition bias; one study had reporting bias | HIC: 10  MIC: 3 | EDN – Dsg, Cnt; LRN |
| 2019, Vitale  Italy [96] | To summarize evidence on the value of virtual simulators to improve the acquisition of hysteroscopic skills for experienced and novice surgeons | Pilot studies, pre/post-test studies | Hysteroscopy | In-service: Novice or experience surgeons  N=250 | Simulation – digital psychomotor skills trainer (virtual reality) | Traditional education | Learners: Improvement in novice and experienced surgeons from pre/post-test scores. Attitudes towards the intervention (acceptance of the simulators) were not measured. | Not reported | HIC: 6 | EDN – Dsg, Cnt; LRN |
| 2019, Wahabi  Saudi Arabia [100] | To evaluate the effectiveness of offline computer-based digital education in improving medical doctors’ knowledge, skills and patient-related outcomes | Randomized controlled trials | Health professions education | In-service: Medicine residents, interns practising doctors, dentists  N=1690 | Non-simulation – offline digital education | Traditional education, other offline digital education or no intervention | Learners: Offline computer-based digital education may improve doctors’ knowledge when compared with no intervention, but it’s general impact on doctors’ knowledge and cognitive skill is uncertain.  Patients: Little or no effect in improving patients’ outcomes. | GRADE: Low or very low quality evidence for knowledge, cognitive skills gain and patient outcomes | HIC: 24  MIC: 5 | EDN – Dsg, Cnt; LRN |
| 2019, Xu  Singapore [71] | To assess the effectiveness of digital education to train health professionals in dermatology | Randomized controlled trials | Dermatology | Pre-service: Medical students or students;  In-service: Physicians, nurses  N=955 | Non-simulation – online or offline digital education | Traditional education or no intervention | Learners: The effectiveness of digital education to deliver dermatology training is mixed and the overall findings are inconclusive. Attitude was not evaluated in the studies. | GRADE: Very low quality evidence for knowledge and satisfaction; low quality evidence for skills | HIC: 9  MIC: 3 | EDN – Dsg, Cnt; LRN |
| 2019, Zhang  Singapore [106] | To evaluate the effectiveness of video-assisted debriefing on learners’ reactions, learning, and behaviour compared with verbal debriefing | Randomized controlled trials, quasi-experimental studies, single group studies | Health professions education | Pre-service: Medical, nursing, dietetic or paramedic students;  In-service: Medical anaesthesia or surgical residents, nurses, midwives  N=1145 | Non-simulation – offline digital education (video-assisted debriefing) | Verbal debriefing | Learners: Video-assisted debriefing was effective to improve learners’ satisfaction, learning experience and performance of health professionals, but did not have an advantage over verbal debriefing on knowledge acquisition. | MERSQI: Overall methodological quality was moderate to high (mean=9.5, range=9.5 to 14.5 for RCTs, 9 to 13.5 for quasi-experimental studies), 9 to 12.5 for quasi-experimental single-group studies size | HIC: 22  MIC: 1 | EDN – Dsg, Cnt, Eng; LRN |
| 2020, Barteit  Germany [98] | To review the implementation and effectiveness of e-learning in low-resources settings, as well as evaluation approaches for medical education | Cross-sectional, , randomized or non-randomized controlled trials | Health professions education | Pre-service: Medical students;  In-service: Residents, physicians  N=12294 | Non-simulation – online or offline digital education, mixed online and offline digital education, mobile and/or computer-based education | Traditional education | Learners: Majority of studies reported the effectiveness of e-learning or positive attitudes towards e-learning, but few studies found no difference between e-learning and face-to-face learning with regards to knowledge and skills. However, overall, e-learning in low- and middle income countries has not met its expected potential. | MERSQI and NOS ratings revealed the low quality of the studies’ evidence for comparability, evaluation instrument validity, study outcomes and participant blinding | MIC: 50  LIC: 1  One study in both MIC and LIC | ENV – Set; EDN – Dsg, Cnt; LRN |
| 2020, Berry  Brazil [108] | To evaluate the effectiveness of technology-enhanced learning methods in periodontics | Randomized controlled trials and crossover studies | Periodontics | Pre-service: Undergraduate dentistry students  N=388 | Non-simulation – online or offline digital education;  Simulation – virtual patient (one study) | Traditional education | Learners: Positive reaction towards the use of technology-enhanced learning in learning in all studies that measured this outcome, with some favouring a combination of technology-enhanced learning and traditional learning methods. Four studies did not find improvements in knowledge gained, while two studies showed improvement in students’ knowledge. Studies that assessed behaviour showed a significant difference on when the virtual patient was applied, but not computer-assisted instruction. | Risk of bias: Overall, 5 studies with moderate ROB, 1 study with high ROB, 1 study with low ROB | HIC: 6  MIC: 1 | EDN – Dsg, Cnt; LRN |
| 2020, Dromey  United Kingdom [46] | To investigate the use of high-fidelity simulation in obstetric ultrasound training | Comparative studies, case control studies | Obstetric ultrasound skills | In-service: Obstetrics and gynecology trainees, doctors, emergency medicine trainees, radiology trainees, certified obstetricians  N=229 | Simulation – digital psychomotor skills trainer (high fidelity simulator) | Traditional education | Learners: Study showed that skills can be acquired, improved and assessed using high-fidelity simulators. Simulation training can be used to equip novice trainees with sufficient skills to perform basic obstetric ultrasound in a clinical environment under direct supervision. However, it was unclear whether the acquired skills lead to improved clinical performance or were retained over time. | MERSQI: Mean±SD = 11.88±1.81, range 9.5 to 15 | HIC: 8 | EDN – Dsg, Cnt; LRN |
| 2020, Foronda  United States [54] | To examine, appraise and synthesize evidence about virtual simulation in nursing education to determine the learning outcomes | Randomized controlled trials, cohort studies, qualitative studies | Nursing education | Pre-service: Nursing students, students from medical, social work, occupational therapy, physician assistant, physical therapy, pharmacy;  In-service: Associate degree, diploma level or graduate nursing students  N=7310 participants from 79/80 studies. | Simulation – digital psychomotor skills trainer (virtual reality), screen-based virtual reality | Traditional education | Learners: Most studies showed that virtual simulation resulted in significant improvement in skills, but knowledge improvement varied in terms of context and settings. There was high student satisfaction, but critical thinking and self-confidence scores were unclear or mixed. Simulation time correlated with greater learning benefits. | Risk of bias determined according to the type of study conducted.  Cohort studies: ROB due to lack of large sample size, lack of identification of confounding factors and lack of reporting confidence intervals.  RCTs: ROB due to failure to identify the effect size, lack of blinding study personnel.  Qualitative studies: ROB due to lack of consideration of researcher and participant relationship and lack of rigour in data analyses. | HIC: 74  MIC: 6 | EDN – Dsg, Cnt, Pdg; LRN |
| 2020, Lapierre  Canada [45] | To evaluate the effectiveness of interprofessional mannequin-based simulation training on teamwork among real teams involved in trauma resuscitation in the emergency department | Quasi-experimental studies, longitudinal studies | Trauma resuscitation | In-service: Surgeons, nurses, allied health workers, emergency medical workers  N=1069 participants from 10/11 studies; one study reported involving participants from 39 trauma cases. | Simulation – digital psychomotor skills trainer (high fidelity mannequin) | No comparison groups | Learners: All studies showed significant improvement in overall teamwork skills or knowledge after training. Scores on the clinical teamwork scale improved during training but declined to baseline one week after simulation training.  Patients: Only one study assessed benefit to the patient – no change in complication, mortality rates observed, or hospital length of stay or intensive care unit length of stay  Institution: Only one study assessed impact. Most of the clinical indicators decreased, showing enhancement of trauma teams’ ability to reach standard critical time indicators with their organisation | JBI Critical Appraisals Checklist for Quasi-experimental studies: Methodological quality ranged from low to moderate | HIC: 11 | EDN – Dsg, Cnt, Evl; LRN |
| 2020, Lee  Republic of Korea [95] | To evaluate virtual patient-based medical communication skills training systems in order to identify features of successful cases | Quantitative and qualitative studies | Medical communication | Pre-service: Medical students;  In-service: Postgraduate residents or fellows  N=1187 | Simulation – virtual patients | Traditional education or no intervention | Learners: The effects of virtual patient training systems on skills outcomes were mixed. Students preferred control conditions (video learning or real patients) over virtual patients. | MERSQI: Quantitative studies score was 13.5 (range 8 to 16.8), which is relatively high;  QualSyst score of the qualitative study was 0.85, which can be interpreted as high quality | HIC: 14 | EDN – Dsg, Cnt, Eng; LRN |
| 2020, Männistö  Finland [104] | To evaluate the effectiveness of digital collaborative learning in nursing education | Randomized controlled trials | Nursing education | Pre-service: Nursing students  N=647 | Non-simulation – online digital education | Traditional education | Learners: Digital collaborative learning improved the development of students’ knowledge, skills and competence, but the outcomes were not all statistically significant. | Joanna Briggs Institute Critical Appraisal Checklist for RCTs: quality score ranged from 9 to 11 (maximum score 13);  The risk of bias was high or unclear for most categories | HIC: 4  MIC: 1 | EDN – Dsg, Cnt, Pdg; LRN |
| 2020, Martinengo  Singapore [55] | To assess the effectiveness and cost-effectiveness of digital education on health professionals’ knowledge, skills, attitudes, satisfaction and behaviour about chronic wound management | Randomized controlled or quasi-randomized trials | Chronic wound management | Pre-service: Nursing students;  In-service: Nurses  N=1404 | Non-simulation – online digital education, offline or offline blended digital education | No intervention or other offline blended digital education modalities | Learners: Digital education alone was less effective than blended digital education but more effective than no intervention in improving knowledge in chronic wound management. Findings for other outcomes were mixed. | Risk of bias: All studies had unclear ROB in ≥2 categories; five studies had high ROB in 1 category | HIC: 6  MIC: 1 | EDN – Dsg, Cnt; LRN |
| 2020, Rangarajan  United Kingdom [72] | To assess the effectiveness of haptics in virtual reality surgical simulation for surgery training | Randomized controlled trials, surveys | Surgery | Pre-service: Medical students;  In-service: Novice or expert surgeons  N=215 | Simulation – digital psychomotor skills trainer (haptics) | Simulator with no haptic feedback | Learners: Mixed results were reported. Training on simulators with haptics improved skills performance across multiple assessment parameters, but evidence regarding the effectiveness was still inconsistent. | Risk of bias: Overall ROB of studies was either moderate or high | HIC: 9 | EDN – Dsg, Cnt, Eng; LRN |
| 2020, Rothschild  United Kingdom [102] | To evaluate whether virtual reality simulation training results in reduced complication rate from real-life cataract surgery | Comparative case series, retrospective comparative cohort or interventional case series, pilot studies | Cataract surgery | In-service: Ophthalmology trainee surgeons, fellows  N=471 | Simulation – digital psychomotor skills trainer (virtual reality) | Non-simulation methods of learning | Learners: Learners skills not directly measured.  Patients: Virtual reality simulation appears to be most helpful in reducing the rate of posterior capsular rupture or errant curvilinear capsulorrhexis.  Institution: It was reported that medical complications and medical negligence claims were reduced (reported in one study). | Risk of bias: All studies had unclear ROB in ≥ 2 categories. One study had ROB in 3 categories | HIC: 8  MIC: 2 | EDN – Dsg, Cnt, Evl |
| 2020, Rourke  United Kingdom [73] | To assess the impact of virtual reality training on the acquisition of clinical psychomotor skills in nursing students | Single group cross-sectional or post-test, single group pre/post-test, non-randomized or randomized controlled trials | Clinical psychomotor skills | Pre-service: Nursing students  N=564 | Simulation – digital psychomotor skills trainer (virtual reality) | Mannequin or part-task trainer | Learners: Post-intervention knowledge and skills improved in the VR group. The skill success rate was improved but showed no significant difference, but the outcome for time to complete the task was mixed. | Centre for Reviews and Dissemination: Mixed methodological quality | HIC: 8  MIC: 1 | EDN – Dsg, Cnt; LRN |
