# Supplementary material for: Digital Education for Health Professionals: An Evidence Map, Conceptual Framework, and Research Agenda
Source: J Med Internet Res. 2022 Mar 17;24(3):e31977. doi: 10.2196/31977 (PMC8972116; doi:10.2196/31977)
Supplement: Multimedia Appendix 4 [file jmir_v24i3e31977_app4.docx]

##### Appendix 4. Research questions identified in the included systematic reviews

| No. | Topic | Question | Digital modality or technology on which the systematic review focuses | | | | | | | | | | References |
| --- | --- | --- | --- | --- | --- | --- | --- | --- | --- | --- | --- | --- | --- |
|  |  |  | Online digital education | Offline digital education | Mobile learning | Digital psychomotor skills trainer | | | | VR/ VRE | Virtual patient | SGG |  |
|  |  |  |  |  |  | Video box model simulator | Virtual reality simulator | High fidelity mannequin | High fidelity simulation |  |  |  |  |
|  | CON-Socio-cult | How do cost and cost-related outcomes influence the adoption of digital technology in health professions education? | √ | √ | √ |  | √ |  |  |  |  |  | [41, 59, 66-73] |
|  | CON-Socio-cult | How can policymakers be incentivized to adopt digital education as part of health professions education? | √ | √ |  |  |  | √ |  | √ |  |  | [59, 74] |
|  | CON-Socio-cult | How do cultural factors within different countries determine the use of digital education for health professions training? |  | √ |  |  |  |  |  |  |  |  | [74] |
|  | CON-Socio-cult | How does providing access to digital education improve the learning outcomes of health professionals? | √ | √ | √ |  | √ |  | √ | √ | √ | √ | [14, 40, 41, 43, 46, 53, 59, 60, 74-85] |
|  | CON-Inst | What is the long-term cost-effectiveness of digital education compared to traditional education for health professionals? | √ |  |  |  | √ |  | √ | √ |  | √ | [12-14, 16, 47, 69, 86] |
|  | CON-Inst | How does health professions digital education impact individual and health services outcomes, and organizational practice? | √ | √ | √ |  | √ | √ |  | √ | √ |  | [11-14, 16, 32, 34, 40, 42-45, 48, 49, 51-53, 59, 68, 70, 72, 75-78, 83, 87-96] |
|  | CON-Inst | Is health professions digital education more time-efficient than traditional education? |  |  |  |  |  |  | √ |  |  |  | [46] |
|  | CON-Set | What is the feasibility of implementing digital technology for health professions education in different socioeconomic settings? | √ | √ |  |  | √ | √ |  | √ |  | √ | [13, 14, 16, 43, 59, 60, 86, 97, 98] |
|  | CON-Set | What are the short- and long-term effects of using digital technology for health professions education in different socioeconomic settings? | √ | √ | √ | √ | √ | √ |  | √ | √ | √ | [32, 43, 47, 50, 54, 68, 86, 90, 97-99] |
|  | CON-Set | Is digital education for health professionals effective in different socioeconomic settings? | √ | √ |  |  |  |  |  |  | √ |  | [13, 40, 99, 100] |
|  | CON-Set | What are the resource requirements to implement digital education in different socioeconomic settings? | √ | √ |  |  |  | √ |  |  |  | √ | [93, 101] |
|  | CON-Set | What are the challenges of setting up digital education for health professionals training in different socioeconomic settings? | √ | √ |  |  |  |  |  |  |  |  | [93] |
|  | CON-Lv | What is the differential impact of digital education on the clinical performance of trainee or expert surgeons? |  |  |  |  | √ |  |  |  |  |  | [102] |
|  | CON-Lv | How can digital education for health professionals be integrated into normal work practices? |  |  | √ |  |  |  |  |  |  |  | [76] |
|  | CON-Lv | How can digital technology be incorporated into current health professions education and training curriculum to improve learning outcomes? | √ | √ |  |  | √ | √ |  | √ |  |  | [42, 46, 47, 54, 69, 70, 86, 98] |
|  | CON-Lv | Is digital education effective in improving health professionals' knowledge and skills performance in the clinical setting? | √ | √ | √ |  | √ | √ |  | √ | √ |  | [11-14, 16, 32, 34, 40, 42-45, 48, 49, 51-53, 59, 68, 70, 72, 75-78, 83, 87-96] |
|  | INF-Dgt | Which features of digital education (e.g. technical features, fidelity, safety, adaptability, etc) affect the learning outcomes of health professions education? | √ |  |  |  |  |  |  |  |  |  | [13, 103] |
|  | INF-Dgt | What are the minimum requirements for the digital technology used to achieve the effectiveness of digital health professions education? | √ | √ |  |  |  |  |  |  |  |  | [93] |
|  | INF-Dgt | What are the technical resources needed to deliver digital education to healthcare professionals? |  |  |  |  |  | √ |  |  |  |  | [69] |
|  | INF-Reg | How should educators delivering digital health education be assessed and accredited? |  |  |  |  |  |  | √ |  |  |  | [47] |
|  | INF-Reg | What are the best practices for the development, evaluation and use of digital health education in health professions education? |  |  |  |  |  |  |  |  |  | √ | [14] |
|  | INF-Reg | Is the use of accreditation-related milestones in digital health education effective? | √ | √ |  |  |  |  |  |  |  |  | [86] |
|  | INF-HR | What digital skills should instructors facilitating digital health education be competent in? |  |  |  |  |  |  | √ |  |  |  | [47] |
|  | INF-HR | How does the digital competence of teachers impact health professions learning outcomes from digital health education? | √ |  |  |  |  |  |  |  |  |  | [104] |
|  | INF-HR | What are the workforce resources needed for health professions digital education? |  |  |  |  |  |  | √ |  |  |  | [69] |
|  | EDN-Mod | What type of instructional design is used in effective digital education of health professions education? | √ | √ |  |  | √ | √ | √ | √ | √ |  | [47, 60, 91, 95, 102] |
|  | EDN-Mod | Which components of digital health education (e.g. interactivity, feedback) contribute to enhanced learning outcomes? | √ | √ |  |  | √ |  |  | √ |  |  | [45, 52, 66, 75, 105] |
|  | EDN-Mod | What is the optimal use of video-assisted debriefing for health professionals' simulation-based training? |  | √ |  |  |  |  |  |  |  |  | [106] |
|  | EDN-Mod | How does the design of digital education interventions (e.g. format and modality used) in health professions education and training curriculum affect learning outcomes? | √ | √ |  |  | √ |  |  | √ |  |  | [34, 42, 53, 72, 82, 86, 101] |
|  | EDN-Mod | Can digital simulation-based training be used to train non-technical skills in health professionals? |  |  |  |  | √ |  |  | √ |  |  | [44, 77] |
|  | EDN-Mod | What is the effectiveness of digital education (mixed or single modality) compared to non-digital education to deliver health professions education? | √ | √ |  |  | √ |  |  | √ |  |  | [42, 79, 106] |
|  | EDN-Mod | Can digital education complement (i.e. blended) or substitute traditional education for health professionals? | √ | √ |  |  | √ |  |  | √ | √ |  | [54, 77, 107, 108] |
|  | EDN-Mod | Does digital simulation-based psychomotor skills training provide any benefit to the medical trainee? |  |  |  | √ |  |  | √ |  |  |  | [46] |
|  | EDN-Cnt | What are the barriers to obtaining standardized digital education materials for health professions education training and how can they be overcome? |  | √ |  |  |  |  |  |  |  |  | [74] |
|  | EDN-Cnt | What content should be included in debriefing (e.g. digital data) following simulation-based education to achieve improved clinical outcomes? |  |  |  |  | √ |  |  | √ |  |  | [47] |
|  | EDN-Cnt | Can digital education be used to overcome challenges in delivering content-specific topics for health professions education (e.g. surgical training in rare pathologic states)? | √ | √ |  |  | √ |  |  | √ |  |  | [92, 97] |
|  | EDN-InstDsg | Can digital education be designed to achieve learning outcomes denoted in the Kirkpatrick model? | √ | √ |  |  |  |  |  |  |  |  | [109] |
|  | EDN-InstDsg | What learning theories can be used to inform the development of effective digital health professions education? | √ | √ |  |  |  | √ |  |  |  | √ | [13, 14, 55, 67, 71, 90, 101] |
|  | EDN-InstDsg | Is mastery learning via digital education more or as effective as traditional education in terms of clinical psychomotor skills improvement? |  |  |  | √ | √ | √ |  | √ |  |  | [41, 47, 48, 53, 66-68, 86, 110] |
|  | EDN-InstDsg | Is spacing digital simulation-based training more or as effective as traditional education in clinical psychomotor skills development? |  |  |  | √ | √ | √ |  | √ |  |  |  |
|  | EDN-InstDsg | How does the frequency and duration of digital simulation-based psychomotor skills training impact health professionals skills transfer to the clinical setting? |  |  |  | √ | √ | √ |  |  |  |  |  |
|  | EDN-InstDsg | What is the optimal duration, frequency and intensity of digital health professions education programs to impact the learning and clinical outcomes of health professionals? | √ | √ | √ |  | √ |  |  | √ |  |  | [43, 54, 70, 80, 91, 102, 111] |
|  | EDN-InstDsg | What pedagogy should be used in the digital education of health professionals to improve their knowledge and skills? | √ |  | √ |  |  |  | √ |  |  |  | [11, 14, 42, 103, 112] |
|  | EDN-InstDsg | What is the effectiveness of using digital education to train and assess non-technical skills in healthcare professionals? |  |  |  |  | √ |  |  | √ | √ |  | [79, 95] |
|  | EDN-InstDsg | What is the effectiveness of digital problem-based learning in health professions education? | √ | √ |  |  | √ |  |  | √ | √ |  | [34] |
|  | EDN-Eng | How does the interactivity of digital education programmes affect the learning and clinical outcomes of health professionals? | √ | √ |  |  | √ |  |  | √ |  |  | [53, 70, 88, 99] |
|  | EDN-Eng | What is the minimal level of haptic feedback required in digital simulation-based training programs to improve health professionals' psychomotor skills? |  |  |  |  | √ |  |  |  |  |  | [72] |
|  | EDN-Eng | What are learners' acceptability of digital education with different levels of interactivity? | √ |  |  |  |  |  |  |  |  |  | [85] |
|  | EDN-Assm | Which performance metrics or measurement instrument should be used to assess health professionals’ knowledge, skills, attitudes, satisfaction, and clinical outcomes from digital technology-based training programs? | √ | √ | √ | √ | √ | √ |  | √ | √ |  | [12, 14, 44, 45, 51-53, 68, 70, 72, 75-79, 81-83, 85, 86, 91, 95, 98, 100-103, 110, 111, 113] |
|  | EDN-Assm | What is the ideal approach to assessing health professionals' knowledge, skills, attitudes, satisfaction, and clinical outcomes from digital technology-based education and training programs? | √ | √ | √ | √ | √ | √ |  | √ | √ |  |  |
|  | EDN-Assm | Should the evaluation of digital health education include behaviour and clinical outcomes? | √ | √ | √ | √ | √ | √ |  | √ | √ |  |  |
|  | EDN-Assm | What is the impact of digital simulation-based training on clinical outcomes in the short- and long term? |  |  |  |  | √ | √ |  | √ | √ |  | [79, 114] |
|  | EDN-Assm | How should learning outcomes in the field of digital health professions education be defined and standardized? | √ | √ |  |  |  |  |  |  |  |  |  |
|  | EDN-Assm | How does the use of digital education affect health professionals' clinical decision making at the point of care? |  |  | √ |  |  |  |  |  |  |  | [76] |
|  | LRN | How does health professionals’ prior learning experience influence the topics that will benefit from the use of digital education? |  |  | √ |  |  |  |  |  |  |  | [115] |
|  | LRN | What are health professionals' attitudes towards digital delivery of education and training programs? |  | √ |  |  | √ |  |  | √ |  |  | [16, 73, 93, 99] |
|  | LRN | What are healthcare professionals’ learning needs and can they be met by the use of digital simulation training? |  |  |  |  | √ |  |  | √ |  |  | [44] |
|  | RSC | What are the methodological requirements for studies assessing digital health education? | √ | √ | √ |  | √ | √ |  | √ | √ | √ | [12, 16, 48, 53, 66, 67, 71, 73, 74, 78, 79, 82, 90, 91, 100, 103, 105] |
|  | RSC | How should studies on digital health professions education be reported? | √ | √ | √ |  | √ | √ |  | √ | √ | √ |  |
|  | RSC | How should studies of digital health professions education be designed to ensure the generalizability of their findings across different settings? | √ | √ | √ |  | √ | √ |  | √ | √ | √ |  |
|  | GEN** | What are the barriers and facilitators that affect the continued adoption of digital tools in health professions education? |  |  | √ |  |  |  |  |  |  |  | [76] |

*Research questions were raised for specific digital modalities identified but are applicable to research on digital health education in general.

**: This refers to general research questions that apply to all domains and categories.

Online digital education also includes massive open online courses (MOOCs) and learning management systems (LMS)

SGG: serious games and gamification

VR/VRE: virtual reality, virtual reality environment

**Domain** CONTEXT: Context, INF: Infrastructure; EDN: Education; LRN: Learners; RSC: Research.

**Category** Socio-cult: Socio-cultural norms; Inst – Institutional norms; Set: Settings; Lv: Level of education; Dgt: Digital; Reg: Regulatory; HR: Human resources; Mod: Modality; InstDsg: Instructional design; Cnt: Content; Eng: Engagement; Assm-Assessment.
