# Supplementary material for: Digital Education for Health Professionals: An Evidence Map, Conceptual Framework, and Research Agenda
Source: J Med Internet Res. 2022 Mar 17;24(3):e31977. doi: 10.2196/31977 (PMC8972116; doi:10.2196/31977)
Supplement: Multimedia Appendix 5 [file jmir_v24i3e31977_app5.docx]

##### Appendix 5. Overview of the conceptual frameworks on digital education implementation and adoption

| **No.** | **Framework** | **Authors and year of publication** | **Aim** | **Structure** | **Overlap between the frameworks** | **Additional information not covered in our framework** |
| --- | --- | --- | --- | --- | --- | --- |
| 1. | eLearning for health workforce capacity building | Tudor Car, 2017 [33] | To outline the role of health leadership and management capacity building (traditional and eLearning) in health system outcomes | Three concentric levels at which capacity building takes place – system, organizational and individual level. They are affected by external, contextual factors. | Context, Infrastructure, Education, Learners | The framework concentrated on health management and leadership capacity building at the micro-, meso- and macro-organizational level. |
| 2. | Global e-Learning framework | Khan, 2010 [35] | To enable systematic understanding of factors helping to create meaningful learning environments | Eight dimensions: institutional, management, technological, pedagogical, ethical, interface design, resource support, and evaluation. | Infrastructure (Digital, Human Resources), Education (Design, Pedagogy, Evaluation) | The framework identifies Institutional (administrative and services), Ethical and Management aspects of e-learning. |
| 3. | Holistic e-learning systems theoretical framework | Aparicio, 2016 [36] | To provide the theoretical background for e-Learning research strategies | 3 principal dimensions: users,  technology, and services. The “People” dimension includes customers, suppliers, professionals associations, SIGs, board and shareholders. The “technology” dimension includes content, communication and collaboration. The “services” dimension includes pedagogical models and instructional strategies. | Education, Infrastructure (Digital, Human Resources) | The framework identifies shareholders in Education Ministry and Industry who would be involved as shareholders. |
| 4. | E-Learning in developing countries | Andersson and Gronlund, 2012 [37] | To outline emerging issues for e-learning in developed and developing countries with the aim of guiding practice and research. | Four categories of challenges for e-Learning: individual (student- and teacher-related), course-related (course design and support provided), contextual (organizational, societal and cultural) and technological. | Context, Infrastructure, Education, Learners | The framework identifies challenges within each category that would impact e-learning. |
| 5. | E-quality framework | Masoum and Lindström, 2012 [116] | A tool for virtual institutions to assess and assure their practices and compare their capability to sustainably develop, deploy, and support e-learning environments with other institution | 7 main factors (technological, pedagogical, student, faculty, evaluation instructional, institutional factor) which serve to classify 29 subfactors | Context (Institutional Norms), Infrastructure (Digital, Human Resources), Education (Evaluation, Pedagogy, Design), Learners, Research | The framework further identified specific sub-factors within each category. |
| 6. | TIPEC framework | Ali, Uppal and Gulliver, 2017 [113] | To consolidate barriers impacting the eLearning implementation as reported in the literature | Four conceptual categories, i.e. Technology (T), Individual (I), Pedagogy (P), and Enabling Conditions (EC) encompassing 68 barriers | Context (Level of education), Infrastructure (Digital, Human Resources), Education (Pedagogy), Learners | The framework identified the barriers within each proposed conceptual category. |
| 7. | Strategic Model Of implementing E-Learning | Madar and Willis, 2014 [115] | To present a model for implementation of eLearning in tertiary institutions | Three main components: Materials development and instructional design (focusing mainly on curriculum development, user profile analysis and pedagogical design), Technology and delivery and Governance and finance | Infrastructure (Digital), Education (Content, Design) | The framework identified governance and finance as a bridge between other components and functions as the overall administration of the system. |
| 8. | An Integrated E-learning Culture Model (IECM) & An e-learning comparative alignment framework (ECAF) | Newton and Ellis, 2007 [117] | To enable understanding e-learning effectiveness factors from multiple perspectives across an organization | Four main factors: the organizational priorities, the learning environment, the instructors’ role and learners’ needs factors. Four interactions between the main factors that influence the effectiveness of eLearning: sustainable e-learning, effective learning opportunities, effective learning and effective training. | Context (Institutional Norms), Infrastructure (Digital, Human Resources), Learners | The framework identifies areas of alignment or misalignment between the main factors. |
| 9. | A framework for the integration of e-learning in higher education institutions in developing countries | Kituyi and Tsubira, 2013 [114] | To guide the integration of eLearning in Higher Education Institutions in developing countries | The framework differentiates activities and relevant stakeholders at three stages: before, during and after the integration. | Context, Infrastructure | The framework specifies what needs to be evaluated at different stages of integration in detail and also includes the private sector as a relevant stakeholder. |
